# Non-Pharmacological Interventions for Return to Work and Multidimensional Health-Related Quality of Life in Adults with Cancer: A Systematic Review and Meta-Analysis of Randomised Trials

**DOI:** 10.3390/healthcare14183128

**Published:** 2026-09-21

**Authors:** Yaning Chen, Yawen Ju, Jiaying Lin, Yuanqing Han, Xinyang Wang, Xintong Luo, Peizhong Peter Wang, Yun Zhu

**Affiliations:** 1 Department of Epidemiology and Biostatistics, School of Public Health, Tianjin Key Laboratory of Environment, Nutrition and Public Health, Tianjin Medical University, Tianjin 300203, China; 2 Department of Epidemiology and Biostatistics, School of Public Health, Hebei Medical University, Shijiazhuang 050017, China; 3 Division of Population Health and Applied Health Sciences, Faculty of Medicine, Memorial University of Newfoundland, St. John’s, NL A1B 3V6, Canada; 4 Centre for New Immigrant Well-Being, Toronto, ON M3B 2R7, Canada; 5 Dalla Lana School of Public Health, University of Toronto, Toronto, ON M5T 3M7, Canada

**Keywords:** cancer survivorship, exercise, health-related quality of life, non-pharmacological intervention, psychoeducation, return to work, meta-analysis, multidisciplinary, randomised controlled trial, rehabilitation

## Abstract

**Highlights:**

**What are the main findings?**

- Exercise, psychoeducational, and multidisciplinary interventions improved overall HRQoL, while exercise showed the broadest benefits across HRQoL domains and psychoeducational interventions showed more targeted improvements in emotional and physical functioning.
- Psychoeducational and multidisciplinary interventions improved return to work; exercise showed a favourable but uncertain effect, whereas vocational interventions showed no clear benefit.

**What are the implications of the main findings?**

- Cancer rehabilitation should be goal-directed and tailored to individual needs, with exercise prioritised for broad functional recovery and psychoeducational support for emotional adjustment and occupational reintegration.
- Rehabilitation may need to integrate physical, psychological, and workplace support for patients facing multiple barriers to recovery, while further trials are needed to clarify the effects of vocational interventions and multidisciplinary approaches on RTW.

**Abstract:**

**Background:** Improving health-related quality of life (HRQoL) and return to work (RTW) among adults with cancer are central to cancer survivorship care, but the effects of different non-pharmacological interventions remain unclear. We evaluated exercise, psychoeducational, vocational, and multidisciplinary interventions across RTW and overall and domain-specific HRQoL. **Methods:** PubMed/MEDLINE, Embase, and CENTRAL were searched from inception to 3 June 2026 for randomised controlled trials reporting HRQoL or RTW. Random-effects models were used to pool standardised mean differences (SMDs) for HRQoL and odds ratios (ORs) for RTW. Risk of bias and evidence certainty were assessed using RoB 2 and GRADE. The protocol was registered in PROSPERO (CRD420261432617). **Results:** Eighty-one randomised controlled trials involving 11,257 participants were included; 67 assessed HRQoL, 17 assessed RTW, and 3 included both outcomes. Of the 67 HRQoL trials, 39 (58%) enrolled exclusively female participants. Compared with usual care, overall HRQoL improved with psychoeducational (SMD, 0.40; 95% CI, 0.16–0.65), exercise (SMD, 0.38; 95% CI, 0.26–0.50), and multidisciplinary interventions (SMD, 0.39; 95% CI, 0.23–0.55), whereas vocational interventions showed no clear benefit (SMD, −0.09; 95% CI, −0.43–0.25). Exercise was associated with improvements across all six evaluable HRQoL domains. Psychoeducational interventions improved emotional and physical functioning, while multidisciplinary interventions produced a small improvement in physical functioning. For RTW, psychoeducational interventions were associated with higher odds of returning to work (OR, 1.41; 95% CI, 1.15–1.73), as were multidisciplinary interventions (OR, 2.26; 95% CI, 1.15–4.48), although the latter estimate was based on only three trials. Exercise interventions also showed a favourable estimate, although the confidence interval included the null (OR, 1.95; 95% CI, 0.99–3.87), whereas vocational interventions showed no clear benefit (OR, 1.29; 95% CI, 0.81--2.08). **Conclusions:** The effects of non-pharmacological rehabilitation differed by intervention type and recovery outcome. Exercise provided the broadest HRQoL benefits, whereas psychoeducational interventions showed the clearest benefits for emotional functioning and RTW. These findings support goal-directed, needs-based rehabilitation and may inform clinical and policy decisions on the delivery of psychological, physical, and workplace support in cancer survivorship care.

## 1. Introduction

Advances in early detection and multimodal treatment have substantially improved cancer survival, resulting in a growing population of working-age adults living beyond cancer [1]. However, many survivors continue to experience long-term physical and psychological sequelae after treatment, which may impair functional recovery and occupational reintegration and increase the risk of unemployment or premature labour-market exit [2,3]. Return to work (RTW) and health-related quality of life (HRQoL) have consequently emerged as complementary indicators of long-term recovery after cancer, with HRQoL capturing multidimensional physical, psychological, and social well-being, and RTW representing occupational recovery and reintegration [4].

Non-pharmacological interventions, including psychoeducational programmes, vocational support, exercise, and multidisciplinary rehabilitation, have been developed to improve employment outcomes and well-being among cancer survivors. However, their effectiveness for RTW remains uncertain. The most recent Cochrane review found that physical and multidisciplinary interventions increased RTW rates, with pooled risk ratios of 1.23 (95% confidence interval [CI], 1.08–1.39) and 1.23 (95% CI, 1.09–1.33), respectively, whereas psychoeducational interventions showed little or no benefit and evidence for vocational interventions was highly uncertain [5]. Other recent systematic reviews focusing on prognostic factors for return to work have similarly highlighted the complexity of occupational reintegration, with available support strategies demonstrating variable and generally modest effects, largely due to substantial clinical and methodological heterogeneity across studies [2,3]. The comparative effectiveness of different non-pharmacological approaches to RTW therefore remains poorly defined.

Evidence for HRQoL is more extensive but remains heterogeneous across intervention types and outcomes. Exercise-based interventions have shown consistent but small improvements in overall HRQoL and related outcomes such as fatigue, dyspnoea, and psychological well-being, with recent meta-analytic evidence reporting standardized mean differences around 0.29 in cancer populations [6], and broader umbrella reviews further supporting benefits across physiological, psychological, and social domains, although certainty varies by outcome [7]. Psychoeducational and psychological interventions also appear beneficial, particularly for reducing anxiety and depressive symptoms and improving selected aspects of quality of life; however, effect sizes are variable and substantial between-study heterogeneity has been consistently observed across different intervention formats and patient groups [4,8]. Lifestyle-related interventions demonstrate potential small-to-moderate improvements in global HRQoL, but findings remain limited by heterogeneity in intervention intensity, adherence, and follow-up duration [9].

Importantly, HRQoL is a multidimensional construct encompassing physical, role, emotional, social, and cognitive functioning, as well as symptom-related domains such as fatigue and vitality. These domains are assessed separately by widely used instruments, including the European Organisation for Research and Treatment of Cancer Quality of Life Questionnaire Core 30 (EORTC QLQ-C30) and the 36-Item Short Form Health Survey (SF-36) [10,11]. Evidence suggests that intervention effects on HRQoL may be domain-specific. Exercise-based interventions have been associated with improvements in physical functioning and fatigue-related outcomes [6,12], whereas psychological and behavioural interventions have shown beneficial effects on overall HRQoL and related psychological outcomes [13,14]. However, most existing meta-analyses have focused on single intervention types or overall HRQoL scores, including recent reviews of digital psychological interventions [8], limiting direct comparison across intervention modalities. Consequently, the comparative effects of psychoeducational, vocational, exercise-based, and multidisciplinary interventions on specific HRQoL domains remain unclear.

We therefore conducted a systematic review and meta-analysis of randomised controlled trials to synthesise the effects of exercise-based, psychoeducational, vocational, and multidisciplinary interventions on RTW, overall HRQoL, and specific HRQoL domains among cancer survivors. By identifying outcome-specific patterns of benefit, this study aimed to support targeted rehabilitation decisions and inform the delivery of cancer survivorship services.

## 2. Methods

### 2.1. Reporting and Registration

This systematic review and meta-analysis followed the Preferred Reporting Items for Systematic Reviews and Meta-Analyses (PRISMA) statement (Supplementary Table S1) [15]. The protocol was prospectively registered with PROSPERO (CRD420261432617). No deviations from the preregistered protocol occurred during the conduct of this review.

### 2.2. Search Strategy and Study Selection

PubMed/MEDLINE, Embase, and the Cochrane Central Register of Controlled Trials (CENTRAL) were searched from inception to 3 June 2026. The search combined terms for cancer and adults with terms related to exercise or physical activity, vocational rehabilitation, psychoeducation, multidisciplinary care, HRQoL, and RTW. Search strategies were informed by previous systematic reviews [4,5,9,16,17]. The complete database-specific strategies are provided in Supplementary Table S2.

Two reviewers independently screened titles and abstracts and assessed potentially eligible full texts. Disagreements were resolved by discussion or, when necessary, adjudication by a third reviewer.

### 2.3. Eligibility Criteria

Randomised controlled trials were eligible if they: (1) enrolled adults aged 18 years or older with a histologically or clinically confirmed solid tumour of any stage who were receiving or had completed primary anticancer treatment; (2) evaluated an exercise, vocational, psychoeducational, or multidisciplinary intervention; (3) used usual care, a waiting-list control, or no intervention as the comparator; (4) reported HRQoL and/or RTW outcomes; and (5) were published in English in a peer-reviewed journal. Studies were excluded if they: (1) included ineligible populations (e.g., patients with haematological malignancies); (2) evaluated ineligible interventions; (3) had ineligible study designs (e.g., non-randomised, single-arm, cross-sectional, qualitative, or crossover studies without extractable first-period data); (4) were protocols, systematic reviews, or meta-analyses; or (5) lacked sufficient numerical data for effect estimation. Exercise interventions included aerobic, resistance, flexibility, yoga, tai chi, and other structured physical activity programmes. Vocational interventions included RTW counselling, workplace accommodation, and occupational rehabilitation. Psychoeducational interventions included cognitive behavioural therapy, mindfulness-based stress reduction, meaning-centred therapy, supportive–expressive therapy, stress management, and cancer-related education. Multidisciplinary interventions combined at least two eligible intervention components. Diet-only interventions were excluded, whereas dietary components delivered alongside an eligible exercise, psychoeducational, or vocational component were classified as multidisciplinary interventions.

Eligible HRQoL outcomes were measured using validated multidimensional instruments, including the global health status/QoL score of the EORTC QLQ-C30 [10,18], the Functional Assessment of Cancer Therapy–General total score (FACT-G) [19], or relevant global or summary measures from the SF-36 or SF-12 [11,20]. Prespecified domain-specific outcomes were physical, role, emotional, social, and cognitive functioning, fatigue, and vitality. RTW outcomes encompassed different operationalisations of occupational recovery, including return to paid employment, current or sustained employment, cessation or duration of sickness absence, work participation or working hours, and self-reported work ability. As these measures capture related but distinct aspects of occupational reintegration, the specific RTW definition used in each study was extracted and reported. Studies of haematological malignancies were excluded. Studies combining participants undergoing treatment with those who had completed treatment were also excluded unless results were reported separately. Other exclusions were non-randomised, single-arm, cross-sectional, qualitative, and uncontrolled before–after studies; study protocols; systematic reviews or meta-analyses; grey literature; and crossover trials without extractable first-period parallel-group data.

### 2.4. Data Extraction

Two reviewers independently extracted the first author, publication year, cancer type, intervention category, outcome instrument, assessment time point, and group-specific outcome data. For HRQoL outcomes, sample sizes, means, and standard deviations (SDs) at the first post-intervention assessment were extracted. Corresponding subscale data were extracted for domain-specific analyses. For binary RTW outcomes, the number of participants returning to work and the total number assessed were recorded; means, SDs, and sample sizes were extracted for continuous RTW outcomes.

For multi-arm trials with a shared control group, the control sample size was divided equally across relevant comparisons, while its mean and SD remained unchanged. When a group mean was reported with a 95% confidence interval (CI), the standard error was calculated as (SE = (upper limit-lower limit)/(2 × 1.96), and the SD as (SD = SE × √n). Studies reporting only graphical data or medians with interquartile ranges were not included in the corresponding quantitative synthesis.

### 2.5. Risk-of-Bias Assessment

Two reviewers (Y.C. and Y.J.) independently assessed risk of bias using the Cochrane Risk of Bias tool 2 (RoB 2) [21]. The assessment covered the randomisation process, deviations from intended interventions, missing outcome data, outcome measurement, and selection of the reported result. Each domain and the overall judgement were classified as low risk, some concerns, or high risk. Overall risk was considered low when all domains were rated as low risk and high when at least one domain was rated as high risk; all other results were classified as having some concerns. Disagreements were resolved by discussion. Certainty of evidence for the primary outcomes was assessed using the GRADE approach, considering risk of bias, inconsistency, indirectness, imprecision, and publication bias, and was rated as high, moderate, low, or very low.

### 2.6. Statistical Analysis

Analyses were conducted using R software (version 4.6.0). Because HRQoL instruments differed across studies, effects on overall HRQoL and individual domains were expressed as standardised mean differences with small-sample correction (Hedges’ (g)) and 95% CIs. Outcome directions were harmonised so that positive estimates favoured the intervention. For binary RTW outcomes, odds ratios (ORs) and 95% CIs were calculated. Continuous RTW outcomes were first expressed as standardised mean differences and then converted to log ORs using (logOR, logOR = SMD × 1.814) (5). Effect directions were aligned so that an OR greater than 1 favoured the intervention. Binary and converted continuous estimates were pooled on the log-OR scale and back-transformed for presentation. For studies with zero events in one group, the Haldane–Anscombe correction of 0.5 was applied to all cells. Given anticipated clinical and methodological heterogeneity, all estimates were pooled using DerSimonian–Laird random-effects models.

Separate meta-analyses were conducted for exercise, vocational, psychoeducational, and multidisciplinary interventions. Within each intervention category, overall HRQoL, RTW, and each prespecified HRQoL domain were analysed separately. Statistical heterogeneity was evaluated using Cochran’s (Q) test and the (I^2^) statistic [22], with values of approximately 25%, 50%, and 75% indicating low, moderate, and high heterogeneity, respectively.

Robustness was assessed using leave-one-out analyses and analyses restricted to studies at low overall risk of bias. For outcomes including at least ten studies, potential publication bias was assessed by visual inspection of funnel plots.

## 3. Results

### 3.1. Study Selection

The database search identified 3934 records: 1153 from PubMed/MEDLINE, 1887 from Embase, and 894 from CENTRAL. After removing 774 duplicates, 3160 records underwent title and abstract screening, of which 2988 were excluded. Of the 172 reports sought for retrieval, one was unavailable (conference abstract only), leaving 171 reports for full-text eligibility assessment.

**Figure 1:**
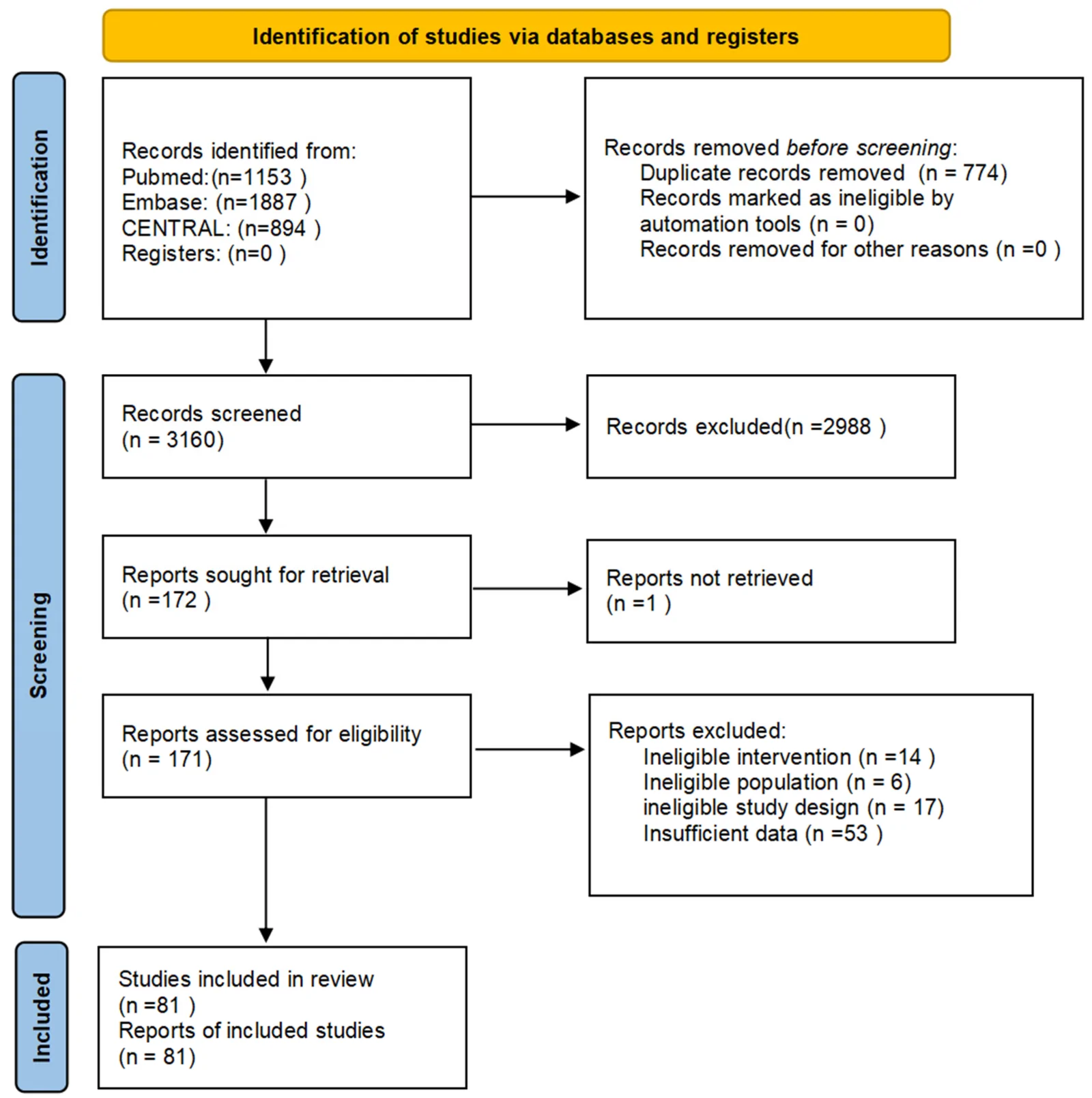
PRISMA flow diagram of included studies.

Of the 171 reports assessed, 90 were excluded: 14 because of ineligible interventions, 6 because of ineligible populations, 17 due to ineligible study designs, and 53 because they lacked sufficient numerical data for effect estimation, such as results presented only graphically or missing SDs or change scores. Overall, 81 studies met the eligibility criteria and were included in the review (Figure 1).

### 3.2. Study Characteristics

The 81 included studies comprised 11,257 participants, including 6450 assigned to intervention groups and 4807 to control groups. Sixty-seven trials reported HRQoL, 17 reported RTW, and 3 reported both outcomes. Individual trial sample sizes ranged from 13 to 1012 participants, and the participant-weighted mean age was 55.7 years.

Exercise interventions involved the largest number of participants (*n* = 4914; 2760 intervention and 2154 control), followed by psychoeducational interventions (*n* = 1845; 1011 and 834, respectively), multidisciplinary interventions (*n* = 1133; 639 and 494), and vocational interventions (*n* = 273; 136 and 137).

Most trials were conducted in the United States (*n* = 16), the Netherlands (*n* = 10), the United Kingdom (*n* = 10), Canada (*n* = 9), South Korea (*n* = 8), or Spain (*n* = 5), with the remainder conducted across Europe, Asia, Australia, the Middle East, North Africa, and the Caribbean. Breast cancer was the most frequently studied malignancy (*n* = 44, 54.3%), followed by colorectal cancer (*n* = 6, 7.4%), prostate cancer (*n* = 8, 9.9%), and mixed or other cancers (*n* = 23, 28.4%).

The Functional Assessment of Cancer Therapy instruments were most frequently used to assess HRQoL, followed by the European Organisation for Research and Treatment of Cancer Quality of Life Questionnaire Core 30 and Short Form Health Survey instruments. RTW was most commonly assessed using self-reported employment status or sickness-absence records. Among studies reporting mode of delivery, supervised face-to-face programmes were most common (*n* = 26), followed by online or remote (*n* = 20) and hybrid programmes (*n* = 15). Detailed study characteristics of the included studies are presented in Table 1 and Table 2.

### 3.3. Overall Health-Related Quality of Life

**Figure 2:**
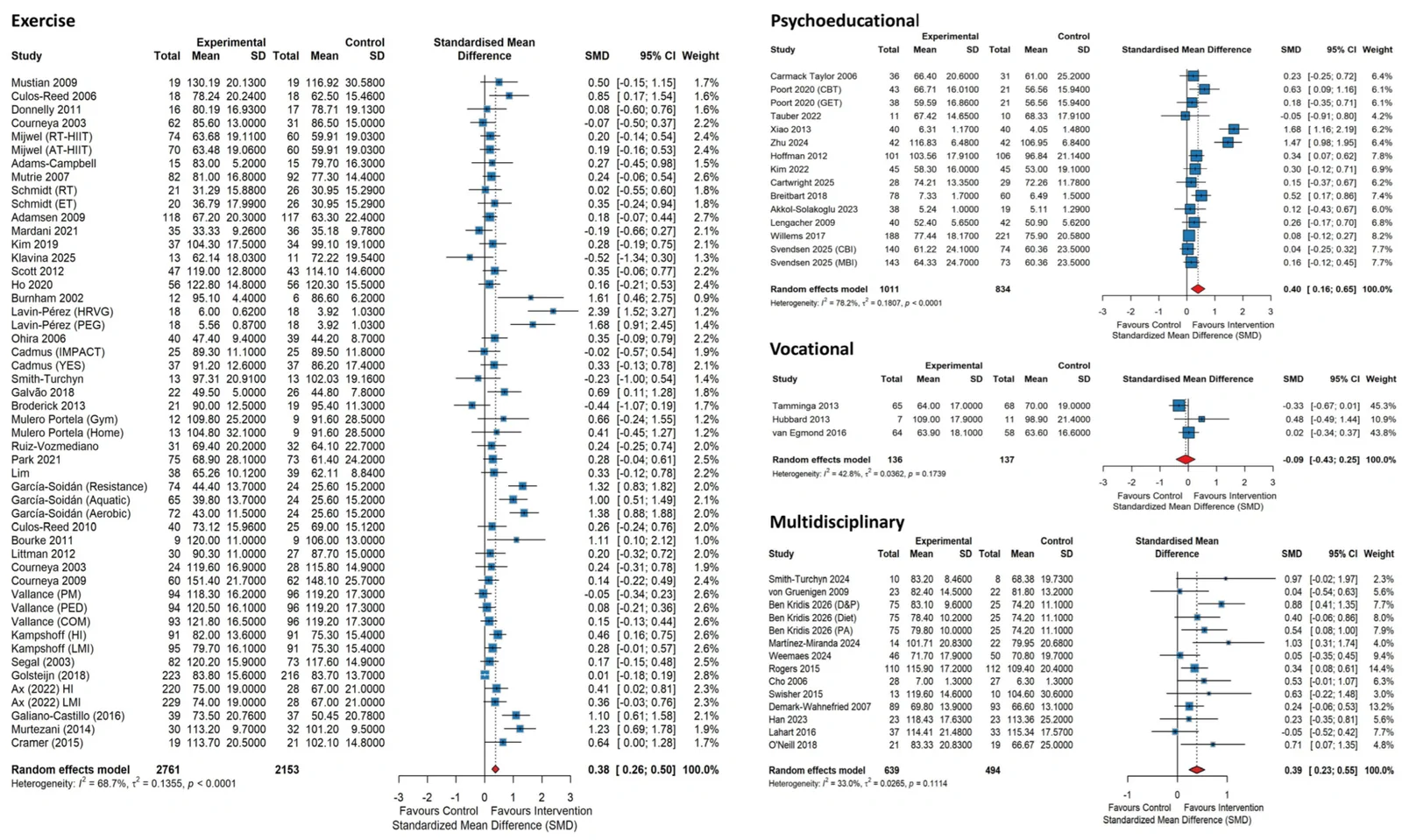
Forest plot for the meta-analysis of overall HRQoL Box size corresponds to each study’s weight in the pooled effect estimate. Blue squares indicate individual study estimates, and red diamonds indicate pooled estimates. Studies included in the forest plot are cited as follows: exercise [23,24,25,26,27,28,29,30,31,32,33,34,35,36,37,38,39,40,41,42,43,44,45,46,47,48,49,50,51,52,53,54,55,56,57,58,59,60,61], psychoeducational [62,63,64,65,66,67,68,69,70,71,72,73,74], vocational [75,76,77], and multidisciplinary interventions [78,79,80,81,82,83,84,85,86,87,88,89]. SD, standard deviation; SMD, standardized mean difference.

At the first post-intervention assessment, 67 studies across the four intervention categories contributed to the HRQoL meta-analyses. Psychoeducational interventions were associated with improved overall HRQoL (SMD, 0.40; 95% CI, 0.16 to 0.65; (I^2^ = 78.2%)) (Figure 2), as were exercise interventions (SMD, 0.38; 95% CI, 0.26 to 0.50; (I^2^ = 68.7%)) and multidisciplinary interventions (SMD, 0.39; 95% CI, 0.23 to 0.55; (I^2^ = 33.0%)). Vocational interventions showed no clear association with overall HRQoL (SMD, −0.09; 95% CI, −0.43 to 0.25; (I^2^ = 42.8%)).

### 3.4. Domain-Specific Health-Related Quality of Life

Six HRQoL domains had sufficient data for quantitative synthesis (Supplementary Figure S1). Exercise interventions were associated with improvements in role functioning (SMD, 0.36; 95% CI, 0.22 to 0.50), physical functioning (SMD, 0.29; 95% CI, 0.18 to 0.39), emotional functioning (SMD, 0.28; 95% CI, 0.10 to 0.45), social functioning (SMD, 0.19; 95% CI, 0.10 to 0.28), and cognitive functioning (SMD, 0.18; 95% CI, 0.01 to 0.34), and with reduced fatigue (SMD, 0.26; 95% CI, 0.19 to 0.34).

Psychoeducational interventions were associated with improvements in emotional functioning (SMD, 0.30; 95% CI, 0.04 to 0.55) and physical functioning (SMD, 0.14; 95% CI, 0.01 to 0.27). The estimate for social functioning favoured psychoeducation but was imprecise (SMD, 0.13; 95% CI, 0.00 to 0.25). Data were insufficient to pool effects on role functioning, cognitive functioning, or fatigue.

Multidisciplinary interventions were associated with a small improvement in physical functioning (SMD, 0.18; 95% CI, 0.01 to 0.36), but not emotional functioning (SMD, 0.22; 95% CI, −0.04 to 0.47), role functioning (SMD, 0.08; 95% CI, −0.19 to 0.35), social functioning (SMD, 0.08; 95% CI, −0.09 to 0.25), or fatigue (SMD, 0.07; 95% CI, −0.45 to 0.59). Evidence was insufficient to pool cognitive functioning.

Vocational interventions were not associated with role functioning (SMD, −0.12; 95% CI, −0.44 to 0.21), social functioning (SMD, −0.11; 95% CI, −0.35 to 0.12), emotional functioning (SMD, −0.06; 95% CI, −0.29 to 0.18), or physical functioning (SMD, −0.04; 95% CI, −0.35 to 0.27). Cognitive functioning, fatigue, and vitality could not be pooled because of insufficient data.

### 3.5. Return to Work

**Figure 3:**
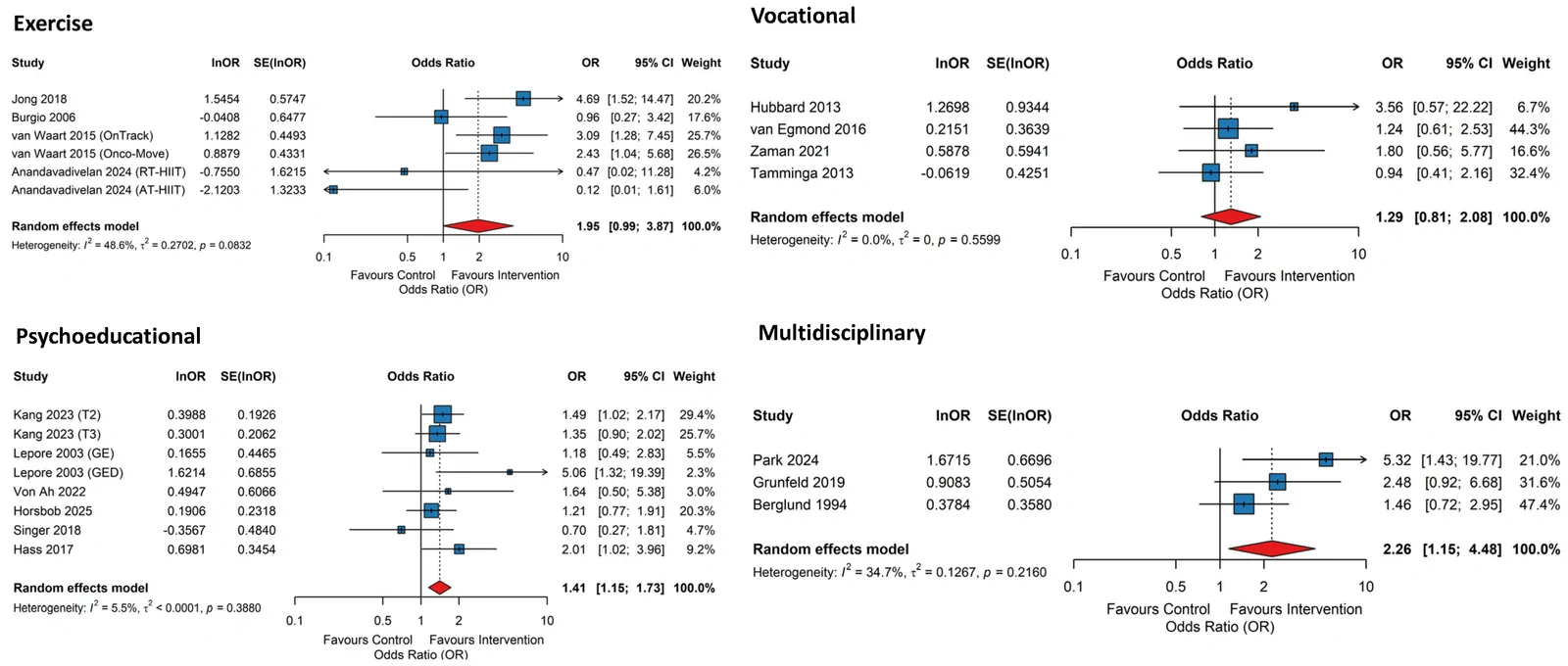
Forest plot for the meta-analysis of return-to-work interventions. Box size corresponds to each study’s weight in the pooled effect estimate. Blue squares indicate individual study estimates, and red diamonds indicate pooled estimates. Studies included in the forest plot are cited as follows: exercise [90,91,92,93], psychoeducational [94,95,96,97,98,99], vocational [75,76,77,100], and multidisciplinary interventions [101,102,103]. SE(lnOR), standard error of log odds ratio; CI, confidence interval.

Seventeen studies contributed to the RTW analyses (Figure 3). Psychoeducational interventions were associated with higher odds of RTW than usual care (OR, 1.41; 95% CI, 1.15 to 1.73; I^2^ = 5.5%). Multidisciplinary interventions also showed higher odds of RTW (OR, 2.26; 95% CI, 1.15 to 4.48; I^2^ = 34.7%), although this estimate was based on only three trials. The pooled estimate for exercise interventions also favoured the intervention, but the 95% CI included the null (OR, 1.95; 95% CI, 0.99 to 3.87; I^2^ = 48.6%). In contrast, vocational interventions were not associated with RTW (OR, 1.29; 95% CI, 0.81 to 2.08; I^2^ = 0.0%).

### 3.6. Risk of Bias and Certainty of Evidence

**Figure 4:**
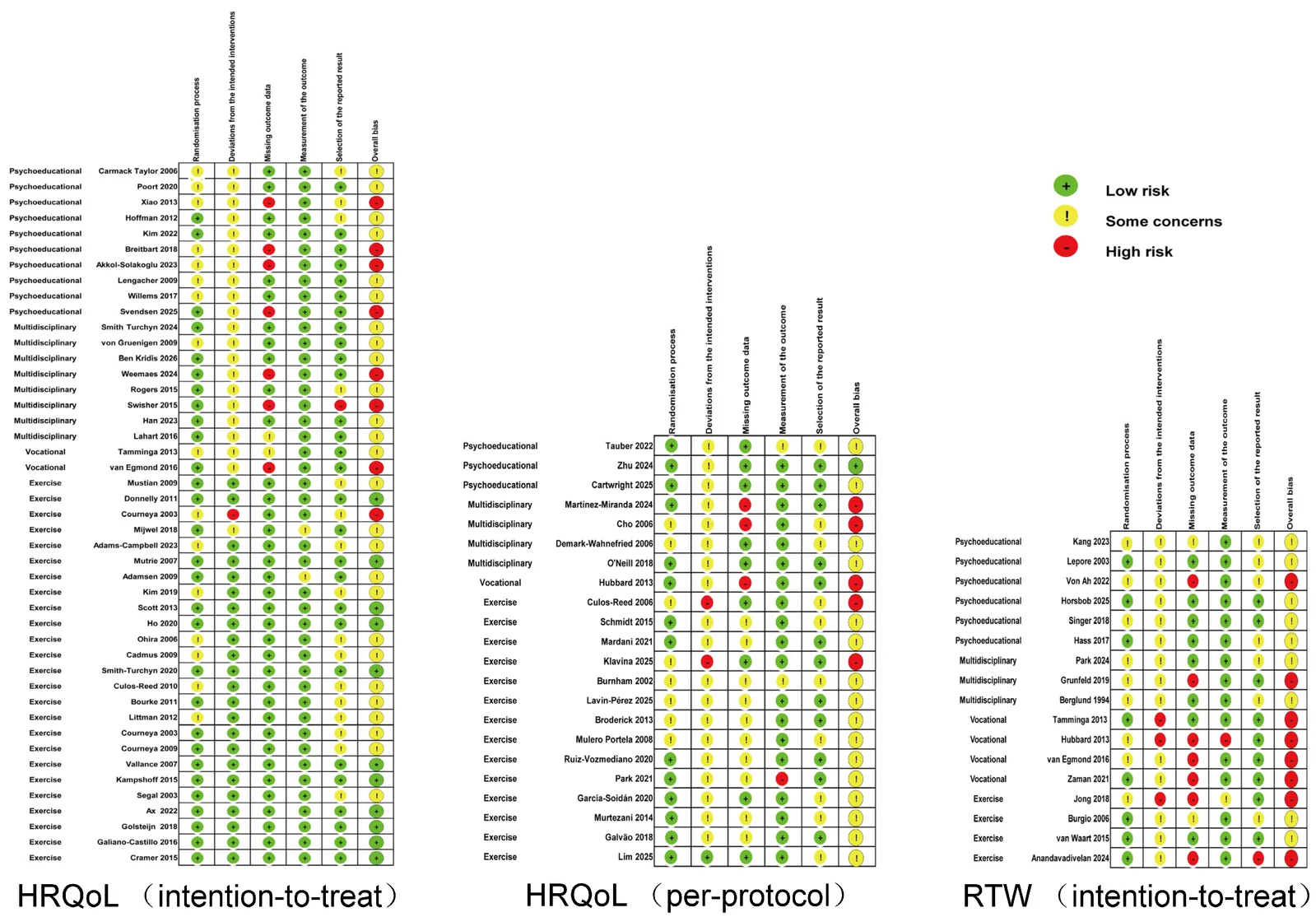
Traffic light plot of ROB 2.0 risk of bias for included studies. References by panel and intervention type are as follows: HRQoL (intention-to-treat), exercise [23,24,25,26,29,30,31,32,33,35,36,37,40,41,42,43,47,49,52,53,54,58,59,60,61], psychoeducational [62,63,64,66,67,68,69,70,72,73], vocational [76,77], and multidisciplinary [78,81,82,85,86,87,88,89]; HRQoL (per-protocol), exercise [27,28,34,38,39,44,45,46,48,50,51,55,56,57], psychoeducational [65,71,74], vocational [75], and multidisciplinary [79,80,83,84]; RTW (intention-to-treat), exercise [90,91,92,93], psychoeducational [94,95,96,97,98,99], vocational [75,76,77,100], and multidisciplinary [101,102,103].

Risk-of-bias assessments for the 81 included randomised controlled trials are summarised in Figure 4. Among the 39 exercise trials reporting HRQoL, 11 (28.2%) were judged to be at low overall risk of bias, the highest proportion among the four intervention categories. Most remaining exercise trials raised some concerns, and none was judged to be at high risk. For RTW, 2 of the 4 exercise trials were at low risk of bias. Trials of psychoeducational, multidisciplinary, and vocational interventions were more commonly rated as having some concerns, largely because of limitations in allocation procedures, deviations from intended interventions, missing outcome data, or outcome measurement.

The certainty of evidence varied across intervention categories and outcomes. Evidence for vocational interventions was rated as low to very low for both HRQoL and RTW, primarily because of the small number of trials, imprecision, and methodological limitations. Detailed risk-of-bias assessments and GRADE ratings are presented in Figure 4 and Supplementary Table S3.

### 3.7. Sensitivity Analyses and Reporting Bias

Leave-one-out analyses showed that excluding individual studies did not materially change the direction, magnitude, or statistical significance of the pooled estimates for the principal and domain-specific HRQoL and RTW outcomes (Supplementary Figure S2). Where sufficient studies were available, analyses restricted to trials at low overall risk of bias yielded results consistent with the primary analyses. Funnel plots for outcomes reported by at least 10 studies were broadly symmetrical, with no clear evidence of small-study effects or publication bias (Supplementary Figure S3). Interpretation was limited by the modest number of studies and between-study heterogeneity.

## 4. Discussion

This systematic review and meta-analysis provides a comprehensive assessment of non-pharmacological rehabilitation for HRQoL and RTW among people living with and beyond cancer. Three principal findings emerged. First, exercise, psychoeducational, and multidisciplinary interventions improved overall HRQoL, whereas evidence for vocational interventions remained inconclusive. Second, the pattern of benefit differed across HRQoL domains: exercise produced the broadest improvements, while the effects of psychoeducational interventions were more concentrated in emotional and physical functioning. Third, psychoeducational and multidisciplinary interventions were associated with improved RTW, whereas the effect of exercise interventions remained uncertain despite a favourable point estimate, and vocational interventions showed no clear benefit. These findings suggest that rehabilitation effects are outcome- and intervention-specific rather than uniform across recovery domains.

### 4.1. Health-Related Quality of Life

The observed benefits of exercise and psychoeducational interventions for overall HRQoL are consistent with previous systematic reviews and meta-analyses [6,8,10,13]. More importantly, the domain-specific analyses provide information that is not captured by global HRQoL scores alone.

Exercise was associated with improvements across physical, role, emotional, social, and cognitive functioning, together with reduced cancer-related fatigue. This broad pattern accords with evidence that exercise can counter treatment-related deconditioning, preserve muscle function, and improve fatigue and physical capacity [7,12,104]. Improvements in these proximal outcomes may subsequently facilitate participation in family, social, and occupational roles. Exercise may also influence inflammatory and metabolic pathways, although these mechanisms were not examined directly in the included trials and should therefore be considered explanatory hypotheses rather than demonstrated mediators.

The effects of psychoeducational interventions were more selective, with the clearest improvement observed in emotional functioning. This pattern is consistent with the intended targets of cognitive behavioural therapy, mindfulness-based approaches, stress management, and structured education, which aim to modify illness perceptions, coping responses, and emotional distress [8,13,14]. The smaller improvement in physical functioning may reflect indirect effects mediated through greater confidence, symptom management, or engagement in daily activities. Evidence was insufficient, however, to determine whether psychoeducational interventions consistently affect fatigue, cognition, or role functioning.

Multidisciplinary interventions improved overall HRQoL but showed a clear domain-specific benefit only for physical functioning. Although combining several rehabilitation components could theoretically address multiple survivorship needs, multidisciplinary programmes varied substantially in content, intensity, professional involvement, and delivery. Such heterogeneity may obscure the contribution of individual components. Complex programmes may also increase treatment burden and reduce adherence, thereby attenuating their effectiveness under routine conditions [105].

Vocational interventions were not associated with improved overall or domain-specific HRQoL [3,5]. This may partly reflect their narrower focus on workplace communication, job modification, and employment procedures rather than on persistent physical or psychological symptoms. However, the evidence base was small and of limited certainty [5,106]. The results should therefore not be interpreted as demonstrating that vocational rehabilitation is ineffective, but rather that its effect on HRQoL remains insufficiently established. Moreover, HRQoL may not be the most proximal or sensitive outcome for interventions designed primarily to address employment barriers [106]. Taken together, these domain-specific findings indicate that global HRQoL alone may provide an incomplete account of rehabilitation benefit.

### 4.2. Return to Work

RTW is a complex outcome shaped by physical capacity, psychological readiness, work demands, employer support, social protection systems, and individual expectations. The observed benefit of psychoeducational interventions suggests that psychological and behavioural barriers may be important determinants of occupational reintegration. By improving coping, self-efficacy, problem-solving, and confidence in managing work-related demands, these interventions may facilitate both the decision and the process of returning to employment.

Our findings extend those of the most recent Cochrane review, which reported little or no clear benefit of psychoeducational interventions for RTW [5]. Despite partial overlap in the included studies, the Cochrane search ended in August 2021 and its analysis of psychoeducational interventions for RTW was based on four RCTs, whereas our search extended to June 2026 and incorporated more recent trials, including Kang et al. [96] and Horsbøl et al. [95]. With the addition of these newer data, the pooled effect of psychoeducational interventions became statistically significant, in contrast to the non-significant estimate reported in the Cochrane review. Moreover, the Cochrane review focused primarily on RTW, with QoL as a secondary outcome, whereas our review additionally evaluated overall and domain-specific HRQoL, allowing intervention effects across different dimensions of recovery to be examined.

Exercise interventions produced favourable RTW estimates, but their confidence intervals were wide; multidisciplinary interventions showed a favourable effect (OR, 2.26; 95% CI, 1.15 to 4.48), although based on only three trials. Exercise may support occupational recovery through improvements in fatigue, endurance, and physical function [6,7], while multidisciplinary interventions may address several barriers concurrently [5]. Nevertheless, heterogeneity in intervention content, treatment status, baseline work participation, adherence, and outcome timing may have reduced the precision of these estimates. The absence of statistical significance should therefore be interpreted as uncertainty rather than evidence of no effect.

The lack of a clear benefit from vocational interventions is notable because these programmes directly target employment. Workplace adjustment alone may be insufficient when persistent symptoms, psychological distress, reduced work ability, and uncertainty about role resumption remain unresolved. Their effectiveness may also depend on employer participation, workplace flexibility, labour-market conditions, and national insurance policies [2,5]. These contextual factors are difficult to standardise across trials and may limit the transferability of findings between countries.

The RTW results support a broader conceptualisation of occupational rehabilitation. Interventions may need to combine psychological preparation and functional recovery with workplace-level support rather than addressing these barriers in isolation. However, the present findings do not establish the superiority of any specific multicomponent model, and adequately powered trials are needed to identify the most effective combination and timing of intervention components.

### 4.3. Clinical Implications

The findings support a goal-oriented approach to cancer rehabilitation. Exercise may be particularly appropriate when physical functioning, fatigue, and broader role participation are priority outcomes. Psychoeducational interventions may be more relevant for emotional adjustment and readiness to resume occupational roles. Patients with interacting physical, psychological, and workplace barriers may benefit from coordinated care involving, as appropriate, oncologists, specialist nurses, physiotherapists or exercise professionals, psychologists, occupational therapists, and vocational rehabilitation counsellors, with the composition of the team tailored to individual rehabilitation needs and goals. These results should not be interpreted as a formal ranking of intervention categories. The analyses were based on separate groups of trials rather than direct head-to-head comparisons, and differences in study populations and intervention design may account for part of the variation in pooled estimates. Clinical decisions should therefore consider individual needs, treatment stage, baseline functional status, employment context, feasibility, and patient preference. The optimal timing of these interventions remains uncertain and warrants direct evaluation in future trials.

### 4.4. Strengths and Limitations

A major strength of this review is the integrated evaluation of four intervention categories across occupational recovery, overall HRQoL, and individual HRQoL domains. This approach moves beyond the binary classification of interventions as effective or ineffective and identifies the outcomes for which each strategy may be most relevant. The inclusion of 81 randomised controlled trials and 11,257 participants provided a substantially larger evidence base than previous reviews. Independent study selection, data extraction, and risk-of-bias assessment, together with RoB 2 and GRADE evaluations, strengthened methodological transparency. The stability of the principal findings in leave-one-out analyses further reduced concern that the results were driven by individual studies.

Several limitations warrant consideration. First, substantial heterogeneity was present in several analyses, likely reflecting differences in cancer type, treatment status, intervention characteristics, outcome measurement, and assessment timing. Pooled estimates should therefore be interpreted as average effects across clinically diverse interventions. Second, the evidence base was uneven. Few trials evaluated vocational interventions, RTW, or several HRQoL domains, resulting in imprecise estimates. This was particularly evident for RTW, where the exercise estimate included the null and the multidisciplinary estimate, although significant, was based on only three trials. Third, several trials had methodological limitations related to missing data, outcome measurement, and selective reporting, while blinding was generally infeasible for behavioural and rehabilitation interventions. RTW was also frequently self-reported, which may have introduced measurement error. Fourth, HRQoL was assessed primarily at the first post-intervention time point, leaving the durability of observed benefits uncertain. RTW definitions and assessment periods also varied across studies, and the conversion of continuous work outcomes to a common odds-ratio scale required assumptions that may reduce clinical interpretability. Fifth, generalisability may be limited by the composition and setting of the available evidence. Notably, 39 of the 67 HRQoL trials (58%) enrolled exclusively female participants, limiting the generalisability of these findings to male cancer survivors; more than half of the included trials focused on breast cancer, and most were conducted in high-income countries. Restriction to English-language, peer-reviewed publications may also have introduced language or publication bias. Finally, aggregate data did not permit systematic investigation of potentially important effect modifiers (e.g., cancer stage, treatment type, occupational demands). Future trials should use standardised HRQoL and RTW definitions, report individual functional domains, incorporate longer follow-up, and provide sufficient information to determine which interventions are most effective for particular patient groups and clinical contexts.

## 5. Conclusions

This systematic review and meta-analysis indicates that non-pharmacological rehabilitation can improve recovery outcomes among cancer survivors, although benefits vary by intervention type and therapeutic target. Psychoeducational interventions may be particularly relevant when emotional adjustment and return to work are the primary goals, whereas exercise should be considered when improving physical functioning, reducing fatigue, and enhancing multidimensional HRQoL are priorities. Multidisciplinary interventions may improve overall HRQoL, but their domain-specific and occupational benefits remain uncertain, while evidence for vocational interventions alone is currently insufficient to support their preferential use. Healthcare professionals and policymakers should promote accessible, needs-based rehabilitation pathways that integrate psychological, physical, and workplace support. These conclusions should be interpreted cautiously, as some reports lacked sufficient data for quantitative synthesis. Further well-designed, adequately powered randomised trials with standardised outcomes and longer follow-up are needed to establish the durability and comparative effectiveness of these strategies.

## Figures and Tables

**Table 1:** Characteristics of included studies (HRQoL outcomes).

Author	Year	Country	Cancer Type	Cancer Stage	Treatment Status	Age (Mean ± SD)	Female (%)	Intervention Type	Intervention Format	Assessment Tool	Follow-Up Duration	N (I)	Mean (I)	SD (I)	N (C)	Mean (C)	SD (C)
Adams-Campbell [23]	2023	USA	Breast cancer (African American)	0–III	On-treatment	63.9 ± 3.2	100%	Exercise	Supervised	FACT-B	8 weeks	15	83.00	5.20	15	79.70	16.30
Adamsen [24]	2009	Denmark	Mixed (21 types)	Mixed	On-treatment	47.2 ± 10.6	72.9%	Exercise	Face-to-face group	EORTC GHS	6 weeks	118	67.20	20.30	117	63.30	22.40
Ax (HI) [25]	2022	Sweden	Mixed (breast + colorectal + prostate)	I–III	On-treatment	58.0 ± 12.0	~80%	Exercise	Mixed	EORTC GHS	6 months	220	75.00	19.00	28	67.00	21.00
Ax (LMI) [25]	2022	Sweden	Mixed (breast + colorectal + prostate)	I–III	On-treatment	58.0 ± 12.0	~80%	Exercise	Mixed	EORTC GHS	6 months	229	74.00	19.00	28	67.00	21.00
Bourke [26]	2011	UK	Colon cancer	Dukes A–C	Post-treatment	69.0 ± 7.5	33.3%	Exercise	Mixed	FACT-C	12 weeks	9	120.00	11.00	9	106.00	13.00
Broderick [27]	2013	Ireland	Mixed (72% breast)	I–III	Post-treatment	51.8 ± 9.2	~86%	Exercise	Supervised	FACT-G	8 weeks	21	90.00	12.50	19	95.40	11.30
Burnham [28]	2002	USA	Breast + colon cancer	Not reported	Post-treatment	54.33 ± 8.63	83.3%	Exercise	Supervised	QoL Index	10 weeks	12	95.10	4.40	6	86.60	6.20
Cadmus (IMPACT) [29]	2009	USA	Breast cancer	0–IIIA	On-treatment	55.0 ± 9.1	100%	Exercise	Home-based	FACT-G	6 months	25	89.30	11.10	25	89.50	11.80
Cadmus (YES) [29]	2009	USA	Breast cancer	0–IIIA	Post-treatment	55.0 ± 9.1	100%	Exercise	Supervised	FACT-G	6 months	37	91.20	12.60	37	86.20	17.40
Courneya [30]	2003	Canada	Colorectal cancer	Not reported	Post-treatment	60.28 ± 10.41	~39%	Exercise	Home-based	FACT-G	16 weeks	62	85.60	13.00	31	86.50	15.00
Courneya [31]	2003	Canada	Breast cancer (postmenopausal)	I–IIIA	Post-treatment	58.6 ± 5.9	100%	Exercise	Supervised	FACT-B	15 weeks	24	119.60	16.90	28	115.80	14.90
Courneya [32]	2009	Canada	Lymphoma (HL/NHL)	I–IV	Mixed	52.97 ± 12.42	~39%	Exercise	Supervised	FACT-An	12 weeks	60	151.40	21.70	62	148.10	25.70
Cramer [33]	2015	Germany	Breast cancer	I–III	Post-treatment	49.2 ± 5.9	100%	Exercise	Face-to-face group	FACT-B	12 weeks	19	113.70	20.50	21	102.10	14.80
Culos-Reed [34]	2006	Canada	Breast (85%) + mixed	Not reported	Post-treatment	51.18 ± 10.33	92%	Exercise	Face-to-face group	EORTC GHS	7 weeks	18	78.24	20.24	18	62.50	15.46
Culos-Reed [35]	2010	Canada	Prostate cancer	Not reported	On-treatment	67.6 ± 8.6	0%	Exercise	Mixed	EORTC GHS	16 weeks	40	73.12	15.96	25	69.00	15.12
Donnelly [36]	2011	UK	Gynecological cancer	I–III	Mixed	53.0 ± 10.3	100%	Exercise	Home-based	FACT-G	12 weeks	16	80.19	16.93	17	78.71	19.13
Galiano-Castillo [37]	2016	Spain	Breast cancer	I–IIIA	Post-treatment	49.81 ± 8.97	100%	Exercise	Online (remote)	EORTC GHS	8 weeks	39	73.50	20.76	37	50.45	20.78
Galvão [38]	2018	Australia	Prostate cancer	Advanced (IV)	On-treatment	70.0 ± 8.4	0%	Exercise	Supervised	SF-36 PF	3 months	22	49.50	5.00	26	44.80	7.80
García-Soidán (Strength) [39]	2020	Spain	Breast cancer	Not reported	Post-treatment	63.0 ± 7.0	100%	Exercise	Supervised	SF-36 GH	2 years	74	44.40	13.70	24.33	25.60	15.20
García-Soidán (Aerobic) [39]	2020	Spain	Breast cancer	Not reported	Post-treatment	63.0 ± 7.0	100%	Exercise	Supervised	SF-36 GH	2 years	72	43.00	11.50	24.33	25.60	15.20
García-Soidán (Aqua fitness) [39]	2020	Spain	Breast cancer	Not reported	Post-treatment	63.0 ± 7.0	100%	Exercise	Supervised	SF-36 GH	2 years	65	39.80	13.70	24.33	25.60	15.20
Golsteijn [40]	2018	Netherlands	Prostate + colorectal	Not reported	Mixed	66.47 ± 7.63	~13%	Exercise	Remote (online + paper)	EORTC GHS	6 months	223	83.80	15.60	216	83.70	13.70
Ho [41]	2020	Hong Kong	Colorectal cancer	I–IV	Post-treatment	65.2 ± 10.1	36.8%	Exercise	Mixed	FACT-C	12 months	56	122.80	14.80	56	120.30	15.50
Kampshoff (HI) [42]	2015	Netherlands	Mixed (65% breast)	I–IV	Post-treatment	53.6 ± 11.1	~80%	Exercise	Supervised	EORTC GHS	12 weeks	91	82.00	13.60	91	75.30	15.40
Kampshoff (LMI) [42]	2015	Netherlands	Mixed (65% breast)	I–IV	Post-treatment	53.6 ± 11.1	~80%	Exercise	Supervised	EORTC GHS	12 weeks	95	79.70	16.10	91	75.30	15.40
Kim [43]	2019	South Korea	Colorectal cancer	II–III	Post-treatment	56.23 ± 9.42	~49.3%	Exercise	Home-based	FACT-C	12 weeks	37	104.30	17.50	34	99.10	19.10
Klavina [44]	2025	Latvia	Breast cancer	IIA–IIIC	Post-treatment	49.75 ± 9.18	100%	Exercise	Online (remote)	EORTC GHS	Immediately post	13	62.14	18.03	11	72.22	19.54
Lavin-Pérez (HRVG) [45]	2025	Spain	Breast cancer	Not reported	Post-treatment	50.35 ± 8.01	100%	Exercise	Online (live)	EORTC GHS	16 weeks	18	6.00	0.62	18	3.92	1.03
Lavin-Pérez (PEG) [45]	2025	Spain	Breast cancer	Not reported	Post-treatment	50.35 ± 8.01	100%	Exercise	Online (live)	EORTC GHS	16 weeks	18	5.56	0.87	18	3.92	1.03
Lim [46]	2025	South Korea	Breast cancer	I–III	Post-treatment	49.35 ± 7.50	100%	Exercise	Online (mixed reality)	FACT-B	8 weeks	38	65.26	10.12	39	62.11	8.84
Littman [47]	2012	USA	Breast cancer	0–III	Post-treatment	59.43 ± 7.97	100%	Exercise	Mixed	FACT-G	6 months	30	90.30	11.00	27	87.70	15.00
Mardani [48]	2021	Iran	Prostate cancer	Not reported	Post-treatment	69.88 ± 5.60	0%	Exercise	Mixed	EORTC GHS	12 weeks	35	33.33	9.26	36	35.18	9.78
Mijwel (AT-HIIT) [49]	2018	Sweden	Breast cancer	I–IIIa	On-treatment	53.23 ± 10.27	100%	Exercise	Supervised	EORTC GHS	16 weeks	70	63.48	19.06	60	59.91	19.03
Mijwel (RT-HIIT) [49]	2018	Sweden	Breast cancer	I–IIIa	On-treatment	53.23 ± 10.27	100%	Exercise	Supervised	EORTC GHS	16 weeks	74	63.68	19.11	60	59.91	19.03
Mulero Portela (Gym) [50]	2008	Puerto Rico	Breast cancer	Not reported	Post-treatment	52.94 ± 11.53	100%	Exercise	Supervised	FACT-B	26 weeks	12	109.80	25.20	9	91.60	28.50
Mulero Portela (Home) [50]	2008	Puerto Rico	Breast cancer	Not reported	Post-treatment	52.94 ± 11.53	100%	Exercise	Home-based	FACT-B	26 weeks	13	104.80	32.10	9	91.60	28.50
Murtezani [51]	2014	Kosovo	Breast cancer	I–IIIA	Post-treatment	52.0 ± 11.0	100%	Exercise	Supervised	FACT-B	10 weeks	30	113.20	9.70	32	101.20	9.50
Mustian [52]	2009	USA	Breast + prostate cancer	Not reported	On-treatment	60.0 ± 12.1	71%	Exercise	Home-based	FACIT-F total	6 weeks	19	130.19	20.13	19	116.92	30.58
Mutrie [53]	2007	UK	Breast cancer	0–III	On-treatment	51.5 ± 9.5	100%	Exercise	Face-to-face group	FACT-G	12 weeks	82	81.00	16.80	92	77.30	14.40
Ohira [54]	2006	USA	Breast cancer	0–III	Post-treatment	53.03 ± 8.23	100%	Exercise	Supervised	CARES-SF Global	6 months	39	44.20	8.70	40	47.40	9.40
Park [55]	2021	South Korea	Prostate cancer	Not reported	On-treatment	66.4 ± 7.5	0%	Exercise	Online (app)	EORTC GHS	12 weeks	75	68.90	28.10	73	61.40	24.20
Ruiz-Vozmediano [56]	2020	Spain	Breast cancer	IIA–IIB	Post-treatment	49.81 ± 8.97	100%	Exercise	Face-to-face group	EORTC GHS	6 months	31	69.40	20.20	32	64.10	22.70
Schmidt (ET) [57]	2015	Germany	Breast cancer	Not reported	On-treatment	54.39 ± 10.67	100%	Exercise	Supervised	EORTC GHS	12 weeks	20	36.79	17.99	26	30.95	15.29
Schmidt (RT) [57]	2015	Germany	Breast cancer	Not reported	On-treatment	54.39 ± 10.67	100%	Exercise	Supervised	EORTC GHS	12 weeks	21	31.29	15.88	26	30.95	15.29
Scott [58]	2013	UK	Breast cancer	I–III	Post-treatment	55.75 ± 9.55	100%	Exercise	Mixed	FACT-B	24 weeks	47	119.00	12.80	43	114.10	14.60
Segal [59]	2003	Canada	Prostate cancer	II–IV	On-treatment	68.0 ± 7.7	0%	Exercise	Supervised	FACT-P	12 weeks	82	120.20	15.90	73	117.60	14.90
Smith-Turchyn [60]	2020	Canada	Breast cancer	Not reported	Mixed	71.9 ± 5.7	100%	Exercise	Online (virtual)	FACT-B	Post-chemo	13	97.31	20.91	13	102.03	19.16
Vallance (COM) [61]	2007	Canada	Breast cancer	I–IIIA	Post-treatment	~58 (SD NR)	100%	Exercise	Remote (mail)	FACT-B	12 weeks	93	121.80	16.50	96	119.20	17.30
Vallance (PED) [61]	2007	Canada	Breast cancer	I–IIIA	Post-treatment	~58 (SD NR)	100%	Exercise	Remote (mail)	FACT-B	12 weeks	94	120.50	16.10	96	119.20	17.30
Vallance (PM) [61]	2007	Canada	Breast cancer	I–IIIA	Post-treatment	~58 (SD NR)	100%	Exercise	Remote (mail)	FACT-B	12 weeks	94	118.30	16.20	96	119.20	17.30
Akkol-Solakoglu [62]	2023	Ireland/UK	Breast cancer	Not reported	Post-treatment	47.96 ± 8.62	100%	Psychoeducational	Online (digital)	EORTC single-item	8 weeks	38	5.24	1.00	19	5.11	1.29
Breitbart [63]	2018	USA	Advanced mixed cancer	Advanced (IV)	On-treatment	58.0 ± 11.0	71.7%	Psychoeducational	Face-to-face individual	MQOL	8 weeks	78	7.33	1.70	60	6.49	1.50
Carmack Taylor [64]	2006	USA	Prostate cancer	Not reported	On-treatment	69.2 (SD NR)	0%	Psychoeducational	Face-to-face group	SF-36 GH	6 months	36	66.40	20.60	31	61.00	25.20
Cartwright [65]	2025	UK	Breast cancer	Not reported	Post-treatment	NR (median 50)	100%	Psychoeducational	Online (digital)	EORTC GHS	1 month	28	74.21	13.35	29	72.26	11.78
Hoffman [66]	2012	UK	Breast cancer	0–III	Post-treatment	49.55 ± 9.20	100%	Psychoeducational	Face-to-face group	FACT-B	8 weeks	101	103.56	17.91	106	96.84	21.14
Kim [67]	2022	South Korea	Breast cancer	0–III	Post-treatment	50.1 ± 9.5	100%	Psychoeducational	Online (telephone)	SF-36 GH	8 weeks	45	58.30	16.00	45	53.00	19.10
Lengacher [68]	2009	USA	Breast cancer	0–III	Post-treatment	57.5 ± 9.4	100%	Psychoeducational	Face-to-face group	SF-36 GH	6 weeks	40	52.40	5.65	42	50.90	5.62
Poort (CBT) [69]	2020	Netherlands	Advanced mixed cancer	Advanced/metastatic	On-treatment	62.76 ± 9.35	57%	Psychoeducational	Face-to-face individual	EORTC GHS	14 weeks	43	66.71	16.01	21	56.56	15.94
Poort (GET) [69]	2020	Netherlands	Advanced mixed cancer	Advanced/metastatic	On-treatment	62.76 ± 9.35	57%	Psychoeducational	Face-to-face individual	EORTC GHS	14 weeks	38	59.59	16.86	21	56.56	15.94
Svendsen (CBI) [70]	2025	Norway	Breast cancer	I–III	Post-treatment	52.39 ± 9.40	100%	Psychoeducational	Online (app)	RAND-36 GH	6 months	140	61.22	24.10	74	60.36	23.50
Svendsen (MBI) [70]	2025	Norway	Breast cancer	I–III	Post-treatment	52.39 ± 9.40	100%	Psychoeducational	Online (app)	RAND-36 GH	6 months	143	64.33	24.70	73	60.36	23.50
Tauber [71]	2022	Denmark	Breast cancer	I–III	Post-treatment	52.1 ± 13.2	100%	Psychoeducational	Mixed (individual + group)	EORTC GHS	1 week post	11	67.42	14.65	10	68.33	17.91
Willems [72]	2017	Netherlands	Mixed (71% breast)	Not reported	Post-treatment	56.27 ± 11.15	~81%	Psychoeducational	Online (digital)	EORTC GHS	6 months	188	77.44	18.17	221	75.90	20.58
Xiao [73]	2013	China	Advanced mixed cancer	Advanced	On-treatment	59.16 ± 11.53	~50%	Psychoeducational	Face-to-face individual	Single-item QOL	3 weeks	40	6.31	1.17	40	4.05	1.48
Zhu [74]	2024	China	Non-small cell lung cancer	I–II	Post-treatment	52.27 ± 9.60	55.56%	Psychoeducational	Online (video)	FACT-L	4 weeks	42	116.83	6.48	42	106.95	6.84
Hubbard [75]	2013	UK	Breast cancer	Not reported	Post-treatment	50.5 ± 5.5	100%	Vocational	Mixed	FACT-B	6 months	7	109.00	17.90	11	98.90	21.40
Tamminga [76]	2013	Netherlands	Female cancer (64% breast)	Not reported	On-treatment	47.55 ± 7.99	100%	Vocational	Face-to-face individual	SF-36 GH	12 months	65	64.00	17.00	68	70.00	19.00
van Egmond [77]	2016	Netherlands	Mixed cancer	Not reported	Post-treatment	48.4 ± 8.6	69%	Vocational	Face-to-face individual	EORTC QLQ-C30	12 months	64	63.90	18.10	58	63.60	16.60
Ben Kridis (Diet only) [78]	2026	North Africa	Colorectal cancer	II–III	Post-treatment	59.3 ± 10.6	47.3%	Multidisciplinary	Mixed	FACT-G	12 months	75	78.40	10.20	25	74.20	11.10
Ben Kridis (Dietary + PA) [78]	2026	North Africa	Colorectal cancer	II–III	Post-treatment	59.3 ± 10.6	47.3%	Multidisciplinary	Mixed	FACT-G	12 months	75	83.10	9.60	25	74.20	11.10
Ben Kridis (PA only) [78]	2026	North Africa	Colorectal cancer	II–III	Post-treatment	59.3 ± 10.6	47.3%	Multidisciplinary	Mixed	FACT-G	12 months	75	79.80	10.00	25	74.20	11.10
Cho [79]	2006	South Korea	Breast cancer (early)	I–II	Post-treatment	49.1 ± 7.7	100%	Multidisciplinary	Face-to-face group	Cho VAS	10 weeks	28	7.00	1.30	27	6.30	1.30
Demark-Wahnefried [80]	2006	USA	Breast / prostate cancer	Not reported	Post-treatment	71.7 ± 5.0	57%	Multidisciplinary	Online/remote	FACT-G	6 months	89	69.80	13.90	93	66.60	13.10
Han [81]	2023	South Korea	Breast cancer	I–III	Post-treatment	48.93 ± 7.03	100%	Multidisciplinary	Mixed	FACT-B	12 weeks	23	118.43	17.63	23	113.36	25.20
Lahart [82]	2016	UK	Breast cancer	I–III	Post-treatment	53.55 ± 9.33	100%	Multidisciplinary	Mixed	FACT-B	6 months	37	114.41	21.48	33	115.34	17.57
Martínez-Miranda [83]	2024	Spain	Breast cancer	0–III	Post-treatment	49.65 ± 7.0	100%	Multidisciplinary	Online (interactive group)	FACT-B + 4	12 weeks	14	101.71	20.83	22	79.95	20.68
O’Neill [84]	2018	Ireland	Esophagogastric cancer	Local	Post-treatment	65.61 ± 8.98	18.6%	Multidisciplinary	Mixed	EORTC GHS	12 weeks	21	83.33	20.83	19	66.67	25.00
Rogers [85]	2015	USA	Breast cancer	0–IIIA	Post-treatment	54.4 ± 8.5	100%	Multidisciplinary	Mixed	FACT-B	3 months	110	115.90	17.20	112	109.40	20.40
Smith-Turchyn [86]	2024	Canada	Mixed (61% breast, etc.)	Not reported	Mixed	71.9 ± 5.7	100%	Multidisciplinary	Online (virtual)	EQ-5D-VAS	10 weeks	10	83.20	8.46	8	68.38	19.73
Swisher [87]	2015	USA	Triple-negative breast cancer	I–III	Post-treatment	53.7 (SD NR)	100%	Multidisciplinary	Mixed	FACT-B	12 weeks	13	119.60	14.60	10	104.60	30.60
Weemaes [88]	2024	Netherlands	Mixed (55% breast)	Not reported	Post-treatment	54.1 ± 11.5	~81%	Multidisciplinary	Online (remote)	EORTC GHS	10 weeks + 6mo	46	71.70	17.90	50	70.80	19.70
von Gruenigen [89]	2009	USA	Endometrial cancer	I–II	Post-treatment	NR	100%	Multidisciplinary	Mixed	FACT-G	6 months	23	82.40	14.50	22	81.80	13.20

Abbreviations: I, intervention group; C, control group; SD, standard deviation; NR, not reported. HRQoL measures: EORTC GHS FACT-G/B/C/P/An SF-36 GH EQ-5D-VAS MQOL CARES-SF (see Assessment Tool column for full names). Data are presented as mean (SD) unless otherwise specified.

**Table 2:** Characteristics of included studies (return-to-work outcomes).

Author	Year	Country	Cancer Type & Stage	Female (%)	Intervention Type	N (I)	N (C)	Total N	RTW Definition	Follow-Up	OR (95% CI)
Anandavadivelan (AT-HIIT) [90]	2024	Sweden	Breast cancer I-IIIa	100	Exercise	72	30	102	Self-reported absence of sick leave	5 years	0.12 (0.01–1.79)
Anandavadivelan (RT-HIIT) [90]	2024	Sweden	Breast cancer I-IIIa	100	Exercise	74	30	104	Self-reported absence of sick leave	5 years	0.47 (0.02–11.52)
Burgio [91]	2006	USA	Prostate cancer T1-T3	0	Exercise	57	55	112	Self-reported return to work (retirees excluded)	6 months	0.96 (0.27–3.42)
Jong [92]	2018	Netherlands	Breast cancer I-III	100	Exercise	47	36	83	Semi-structured interview: return to work	6 months	4.69 (1.52–14.46)
van Waart (OnTrack) [93]	2015	Netherlands	Breast cancer I-III	97	Exercise	76	38.5	114.5	Self-reported return to work (questionnaire)	6 months	3.09 (1.28–7.45)
van Waart (Onco-Move) [93]	2015	Netherlands	Breast cancer I-III	100	Exercise	77	38.5	115.5	Self-reported return to work (questionnaire)	6 months	2.43 (1.04–5.68)
Hass [94]	2017	Germany	Breast cancer I-IV	100	Psychoeducational	70	83	153	Successful RTW (<12 weeks absence no disability application)	6 months	2.01 (1.02–3.95)
Horsbøl [95]	2025	Denmark	Breast cancer I-II	100	Psychoeducational	149	139	288	WAS single item (work ability)	36 months	1.21 (0.77–1.91)
Kang (T2) [96]	2023	South Korea	Mixed (60% breast) 0-III	69	Psychoeducational	120	119	239	Currently working (telephone interview)	1 month	1.49 (1.02–2.17)
Kang (T3) [96]	2023	South Korea	Mixed (60% breast) 0-III	69	Psychoeducational	120	119	239	Currently working (telephone interview)	12 months	1.35 (0.90–2.02)
Lepore (GE) [97]	2003	USA	Prostate cancer T1-T3	0	Psychoeducational	84	40	124	Stable employment at 12 months	12 months	1.18 (0.49–2.82)
Lepore (GED) [97]	2003	USA	Prostate cancer T1-T3	0	Psychoeducational	86	40	126	Stable employment at 12 months	12 months	5.06 (1.32–19.39)
Singer [98]	2018	Germany	Mixed cancer	50	Psychoeducational	570	442	1012	Employed (<65 y not retired self-report)	6 months	0.70 (0.30–2.00)
Von Ah [99]	2022	USA	Breast cancer I-IIIa	100	Psychoeducational	19	17	36	WAI single item (work ability)	10 weeks	1.64 (0.50–5.39)
Tamminga [76]	2013	Netherlands	Female cancer (64% breast)	99	Vocational	65	68	133	Full/part-time return to work (self-report)	12 months	0.94 (0.41–2.17)
Hubbard [75]	2013	UK	Breast cancer 0-III	100	Vocational	46	214	260	Adjusted weekly working hours at 12 months	12 months	3.56 (0.57–22.21)
Zaman [100]	2021	Netherlands	GI cancer (curative treatment)	33	Vocational	42	46	88	RTW ≥ 4 weeks (full/part-time self-report)	12 months	1.80 (0.56–5.75)
van Egmond [77]	2016	Netherlands	Mixed (40% breast)	69	Vocational	85	86	171	Sustained RTW ≥ 28 days (registry + self-report)	12 months	1.24 (0.61–2.54)
Berglund [101]	1994	Sweden	Mixed (80% breast)	80	Multidisciplinary	98	101	199	Still on sick leave/not working (self-report)	12 months	1.46 (0.72–2.93)
Grunfeld [102]	2019	UK	Mixed cancer (50% breast)	79	Multidisciplinary	38	30	68	Full/part-time/gradual return to work	12 months	2.48 (0.92–6.67)
Park [103]	2024	South Korea	Colorectal cancer I-IV	28	Multidisciplinary	28	30	58	Return to work (self-report)	12 months	5.32 (1.43–19.74)

Abbreviations: I, intervention group; C, control group; OR, odds ratio; CI, confidence interval. RTW, return to work. Data are presented as OR (95% CI) for return-to-work outcomes.

## Data Availability

No new data were created or analyzed in this study. Data sharing is not applicable to this article.

## Supplementary Materials

The following supporting information can be downloaded at: https://www.mdpi.com/article/10.3390/healthcare14183128/s1: Table S1. PRISMA 2020 Checklist; Table S2. Full search strategies for each database (searched from inception to 3 June 2026); Table S3. GRADE assessment of evidence certainty for primary outcomes; Figure S1. Detailed forest plots of all included studies for overall HRQoL, stratified by intervention type; Figure S2. Leave-one-out sensitivity analyses of pooled intervention effects on overall health-related quality of life (HRQoL) and return-to-work outcomes, stratified by intervention type; Figure S3. Funnel plots for publication bias assessment.
