# Supplementary material for: Non-Pharmacological Interventions for Return to Work and Multidimensional Health-Related Quality of Life in Adults with Cancer: A Systematic Review and Meta-Analysis of Randomised Trials
Source: Healthcare (Basel). 2026 Sep 21;14(18):3128. doi: 10.3390/healthcare14183128 (PMC13606160; doi:10.3390/healthcare14183128)

Supplemental Digital Content

Non-pharmacological Interventions for Return to Work and Multidimensional Health-Related Quality of Life in Adults With Cancer: A Systematic Review and Meta-analysis of Randomised Trials

Supplementary Tables

Table S1. PRISMA 2020 Checklist

Table S2. Full search strategies each database (searched from inception to 3 June 2026)

Table S3. GRADE assessment of evidence certainty for primary outcomes

Supplementary Figures

Figure S1. Detailed forest plots of all included studies for overall HRQoL, stratified by intervention type

Figure S2. Leave-one-out sensitivity analyses of pooled intervention effects on overall health-related quality of life (HRQoL) and return-to-work outcomes, stratified by intervention type

Figure S3. Funnel plots for publication bias assessment

Supplementary Tables

Table S1. PRISMA 2020 Checklist

Section and Topic	Item #	Checklist item	Location where item is reported
**TITLE**			
Title	1	Identify the report as a systematic review.	Title page
**ABSTRACT**			
Abstract	2	See the PRISMA 2020 for Abstracts checklist.	Abstract
**INTRODUCTION**			
Rationale	3	Describe the rationale for the review in the context of existing knowledge.	Introduction (Section 1)
Objectives	4	Provide an explicit statement of the objective(s) or question(s) the review addresses.	Introduction (Section 1, last paragraph)
**METHODS**			
Eligibility criteria	5	Specify the inclusion and exclusion criteria for the review and how studies were grouped for the syntheses.	Methods / Eligibility criteria (Section 2.3)
Information sources	6	Specify all databases, registers, websites, organisations, reference lists and other sources searched or consulted to identify studies. Specify the date when each source was last searched or consulted.	Methods / Search strategy (Section 2.2)
Search strategy	7	Present the full search strategies for all databases, registers and websites, including any filters and limits used.	Supplementary Table S1
Selection process	8	Specify the methods used to decide whether a study met the inclusion criteria of the review, including how many reviewers screened each record and each report retrieved, whether they worked independently, and if applicable, details of automation tools used in the process.	Methods / Study selection (Section

Section and Topic	Item #	Checklist item	Location where item is reported
			2.2)
Data collection process	9	Specify the methods used to collect data from reports, including how many reviewers collected data from each report, whether they worked independently, any processes for obtaining or confirming data from study investigators, and if applicable, details of automation tools used in the process.	Methods / Data extraction (Section 2.4)
Data items	10a	List and define all outcomes for which data were sought. Specify whether all results that were compatible with each outcome domain in each study were sought (e.g. for all measures, time points, analyses), and if not, the methods used to decide which results to collect.	Methods / Data extraction (Section 2.4) – HRQoL (EORTC QLQ-C30, FACT-G, SF-36/SF-12) and RTW outcomes
	10b	List and define all other variables for which data were sought (e.g. participant and intervention characteristics, funding sources). Describe any assumptions made about any missing or unclear information.	Methods / Data extraction (Section 2.4) – first author, year, cancer type, intervention category, assessment tool, time point, sample size, means, SDs; conversions applied where data were missing
Study risk of bias	11	Specify the methods used to assess risk of bias in the included studies, including details of the tool(s) used, how many reviewers assessed	Methods / Risk of

Section and Topic	Item #	Checklist item	Location where item is reported
assessment		each study and whether they worked independently, and if applicable, details of automation tools used in the process.	bias assessment (Section 2.5) – RoB 2 tool, two independent reviewers
Effect measures	12	Specify for each outcome the effect measure(s) (e.g. risk ratio, mean difference) used in the synthesis or presentation of results.	Methods / Statistical analysis (Section 2.6) – SMD (Hedges' g) for HRQoL; OR for binary RTW; converted SMD to logOR for continuous RTW
Synthesis methods	13a	Describe the processes used to decide which studies were eligible for each synthesis (e.g. tabulating the study intervention characteristics and comparing against the planned groups for each synthesis (item #5)).	Methods / Statistical analysis (Section 2.6) – separate meta-analyses by intervention category (exercise, psychoeducational, vocational, multidisciplinary)
	13b	Describe any methods required to prepare the data for presentation or synthesis, such as handling of missing summary statistics, or data conversions.	Methods / Statistical analysis (Section 2.6)

Section and Topic	Item #	Checklist item	Location where item is reported
			– Haldane–Anscombe correction (0.5) for zero events; SE/SD conversions from 95% CIs; continuous RTW converted to logOR using SMD × 1.814
	13c	Describe any methods used to tabulate or visually display results of individual studies and syntheses.	Methods / Statistical analysis (Section 2.6) – forest plots for meta-analyses; tables for study characteristics and individual study results
	13d	Describe any methods used to synthesize results and provide a rationale for the choice(s). If meta-analysis was performed, describe the model(s), method(s) to identify the presence and extent of statistical heterogeneity, and software package(s) used.	Methods / Statistical analysis (Section 2.6) – DerSimonian–Laird random-effects model (anticipating heterogeneity); I^2 statistic and

Section and Topic	Item #	Checklist item	Location where item is reported
			Cochran's Q; R software version 4.6.0
	13e	Describe any methods used to explore possible causes of heterogeneity among study results (e.g. subgroup analysis, meta-regression).	Methods / Statistical analysis (Section 2.6) –Not performed. No subgroup analyses or meta-regression of potential effect modifiers were conducted because study numbers and the distribution of study-level characteristics were insufficient for reliable analyses.
	13f	Describe any sensitivity analyses conducted to assess robustness of the synthesized results.	Methods / Statistical analysis (Section 2.6) – Leave-one-out analyses and analyses restricted to studies at low overall

Section and Topic	Item #	Checklist item	Location where item is reported
			risk of bias were conducted to assess the robustness of the pooled estimates.
Reporting bias assessment	14	Describe any methods used to assess risk of bias due to missing results in a synthesis (arising from reporting biases).	Methods / Statistical analysis (Section 2.6) – visual inspection of funnel plots for outcomes with ≥10 studies
Certainty assessment	15	Describe any methods used to assess certainty (or confidence) in the body of evidence for an outcome.	Methods / Risk of bias assessment (Section 2.5) – GRADE assessment
**RESULTS**			
Study selection	16a	Describe the results of the search and selection process, from the number of records identified in the search to the number of studies included in the review, ideally using a flow diagram.	Results / Study selection (Section 3.1) and Figure 1 (PRISMA flow diagram)
	16b	Cite studies that might appear to meet the inclusion criteria, but which were excluded, and explain why they were excluded.	Results / Study selection (Section 3.1) – 90 reports

Section and Topic	Item #	Checklist item	Location where item is reported
			excluded: ineligible intervention (n = 14), ineligible population (n = 6), ineligible study design (n = 17), and insufficient numerical data for quantitative synthesis (n = 53).
Study characteristics	17	Cite each included study and present its characteristics.	Results / Study characteristics (Section 3.2) and Table 1 (HRQoL) and Table 2 (RTW)
Risk of bias in studies	18	Present assessments of risk of bias for each included study.	Results / Risk of bias (Section 3.6) and Figure 4
Results of individual studies	19	For all outcomes, present, for each study: (a) summary statistics for each group (where appropriate) and (b) an effect estimate and its precision (e.g. confidence/credible interval), ideally using structured tables or plots.	Results – Table 1 (HRQoL: means, SDs by group) and Table 2 (RTW: ORs and 95% CIs); forest plots (Figure 2，3 and

Section and Topic	Item #	Checklist item	Location where item is reported
			FigureS1)
Results of syntheses	20a	For each synthesis, briefly summarise the characteristics and risk of bias among contributing studies.	Results / Results of syntheses (Sections 3.3–3.5) – characteristics and ROB summarised per intervention category
	20b	Present results of all statistical syntheses conducted. If meta-analysis was done, present for each the summary estimate and its precision (e.g. confidence/credible interval) and measures of statistical heterogeneity. If comparing groups, describe the direction of the effect.	Results / Overall HRQoL (Section 3.3), domain-specific (Section 3.4), RTW (Section 3.5) – all include SMD/OR, 95% CI, I^2, p-values; (Figure 2，3 and FigureS1)
	20c	Present results of all investigations of possible causes of heterogeneity among study results.	Not applicable; no subgroup analyses or meta-regression were conducted.
	20d	Present results of all sensitivity analyses conducted to assess the robustness of the synthesized results.	Results / Sensitivity analyses (Section 3.7) –leave-one-out

Section and Topic	Item #	Checklist item	Location where item is reported
			analyses and analyses restricted to studies at low overall risk of bias.
Reporting biases	21	Present assessments of risk of bias due to missing results (arising from reporting biases) for each synthesis assessed.	Results / Sensitivity analyses (Section 3.7) – funnel plots broadly symmetrical, no clear evidence of small-study effects or publication bias
Certainty of evidence	22	Present assessments of certainty (or confidence) in the body of evidence for each outcome assessed.	Results / Risk of bias and certainty (Section 3.6) – evidence certainty varied: vocational interventions rated low to very low; GRADE details in Supplementary Table S2
**DISCUSSION**			
Discussion	23a	Provide a general interpretation of the results in the context of other evidence.	Discussion (Section

Section and Topic	Item #	Checklist item	Location where item is reported
			4.1 and 4.2) – compared with prior Cochrane review and other meta-analyses
	23b	Discuss any limitations of the evidence included in the review.	Discussion / Limitations (Section 4.4) – heterogeneity, uneven evidence base, lack of blinding, self-reported RTW, short follow-up, predominance of breast cancer and high-income countries
	23c	Discuss any limitations of the review processes used.	Discussion / Limitations (Section 4.4) – conversion of continuous RTW outcomes, language restriction, aggregate data precluding effect-modifier

Section and Topic	Item #	Checklist item	Location where item is reported
			analysis
	23d	Discuss implications of the results for practice, policy, and future research.	Discussion / Clinical implications (Section 4.3) and Conclusions (Section 5)
**OTHER INFORMATION**			
Registration and protocol	24a	Provide registration information for the review, including register name and registration number, or state that the review was not registered.	Methods / Reporting and Registration (Section 2.1) – PROSPERO (CRD420261432617)
	24b	Indicate where the review protocol can be accessed, or state that a protocol was not prepared.	Methods / Reporting and Registration (Section 2.1) – protocol registered with PROSPERO, accessible via the registry
	24c	Describe and explain any amendments to information provided at registration or in the protocol.	Not applicable –No amendments were made to the registered protocol

Section and Topic	Item #	Checklist item	Location where item is reported
			(PROSPERO CRD420261432617).
Support	25	Describe sources of financial or non-financial support for the review, and the role of the funders or sponsors in the review.	Funding section – funding from the China Scholarship Council (CSC); the funder had no role in study design, data collection, analysis, interpretation, manuscript preparation, or publication decisions.
Competing interests	26	Declare any competing interests of review authors.	Conflicts of Interest section – all authors declare no conflicts of interest
Availability of data, code and other materials	27	Report which of the following are publicly available and where they can be found: template data collection forms; data extracted from included studies; data used for all analyses; analytic code; any other materials used in the review.	Data Availability Statement –No new data were created or analyzed in this study. Data sharing is not applicable to this

Section and Topic	Item #	Checklist item	Location where item is reported
			article.

Table S2. Full search strategies for each database (searched from inception to 3 June 2026)

Database	Search_Strategy
PubMed	("Neoplasms"[Mesh] OR cancer*[tiab] OR carcinoma*[tiab] OR tumor*[tiab] OR tumour*[tiab] OR neoplasm*[tiab] OR malignan*[tiab]) AND ("Cancer Survivors"[Mesh] OR survivor*[tiab]) AND (("Exercise"[Mesh] OR "physical activity"[tiab] OR "exercise therapy"[tiab] OR aerobic[tiab] OR "resistance training"[tiab] OR "strength training"[tiab] OR yoga[tiab] OR "tai chi"[tiab] OR qigong[tiab] OR walking[tiab]) OR ("psychoeducation"[tiab] OR "psycho-education"[tiab] OR "cognitive behavioral therapy"[tiab] OR "cognitive behavioural therapy"[tiab] OR CBT[tiab] OR mindfulness[tiab] OR "mindfulness-based"[tiab] OR "stress management"[tiab] OR "supportive expressive"[tiab] OR "meaning-centered"[tiab] OR counseling[tiab] OR counselling[tiab]) OR ("Vocational Rehabilitation"[Mesh] OR "occupational rehabilitation"[tiab] OR "return to work"[tiab] OR RTW[tiab] OR "workplace intervention"[tiab] OR "job coaching"[tiab] OR "employment support"[tiab] OR "work-related"[tiab]) OR (multidisciplinary[tiab] OR "multi-disciplinary"[tiab] OR "multi-component"[tiab] OR "multicomponent"[tiab] OR "combined intervention"[tiab] OR "integrated care"[tiab])) AND ("Quality of Life"[Mesh] OR "health-related quality of life"[tiab] OR HRQoL[tiab] OR QoL[tiab] OR "EORTC QLQ-C30"[tiab] OR "FACT-G"[tiab] OR "SF-36"[tiab] OR "SF-12"[tiab] OR "return to work"[tiab] OR RTW[tiab] OR "Vocational Rehabilitation"[Mesh]) AND ("Randomized Controlled Trial"[pt] OR "randomized controlled trials as topic"[Mesh] OR RCT[tiab] OR randomized[tiab] OR randomised[tiab] OR "randomized controlled trial"[tiab] OR placebo[tiab])

Database	Search_Strategy
Embase	('neoplasm'/exp OR cancer:ab,ti OR carcinoma:ab,ti OR tumor:ab,ti OR tumour:ab,ti OR neoplasm:ab,ti OR malignan*:ab,ti) AND ('cancer survivor'/exp OR survivor:ab,ti) AND (('exercise'/exp OR 'physical activity':ab,ti OR 'exercise therapy':ab,ti OR aerobic:ab,ti OR 'resistance training':ab,ti OR 'strength training':ab,ti OR yoga:ab,ti OR 'tai chi':ab,ti OR qigong:ab,ti OR walking:ab,ti) OR ('psychoeducation':ab,ti OR 'psycho-education':ab,ti OR 'cognitive behavioral therapy':ab,ti OR 'cognitive behavioural therapy':ab,ti OR CBT:ab,ti OR mindfulness:ab,ti OR 'mindfulness-based':ab,ti OR 'stress management':ab,ti OR 'supportive expressive':ab,ti OR 'meaning-centered':ab,ti OR counseling:ab,ti OR counselling:ab,ti) OR ('vocational rehabilitation'/exp OR 'occupational rehabilitation':ab,ti OR 'return to work':ab,ti OR RTW:ab,ti OR 'workplace intervention':ab,ti OR 'job coaching':ab,ti OR 'employment support':ab,ti OR 'work-related':ab,ti) OR (multidisciplinary:ab,ti OR 'multi-disciplinary':ab,ti OR 'multi-component':ab,ti OR multicomponent:ab,ti OR 'combined intervention':ab,ti OR 'integrated care':ab,ti)) AND ('quality of life'/exp OR 'health-related quality of life':ab,ti OR HRQoL:ab,ti OR QoL:ab,ti OR 'EORTC QLQ-C30':ab,ti OR 'FACT-G':ab,ti OR 'SF-36':ab,ti OR 'SF-12':ab,ti OR 'return to work':ab,ti OR RTW:ab,ti OR 'vocational rehabilitation'/exp) AND ('randomized controlled trial'/exp OR RCT:ab,ti OR randomized:ab,ti OR randomised:ab,ti OR placebo:ab,ti)
CENTRAL (Cochrane Library)	(cancer* or carcinoma* or tumor* or tumour* or neoplasm* or malignan*) and survivor* and (survivorship or "post-treatment" or "completed treatment" or rehabilitation) and (exercise or "physical activity" or "exercise therapy" or aerobic or "resistance training" or "strength training" or yoga or "tai chi" or qigong or walking or psychoeducation or "psycho-education" or "cognitive behavioral therapy" or "cognitive behavioural therapy" or CBT or mindfulness or "mindfulness-based" or "stress management" or "supportive expressive" or "meaning-centered" or counseling or counselling or "vocational rehabilitation" or "occupational rehabilitation" or "return to work" or RTW or "workplace intervention" or "job coaching" or "employment support" or "work-related" or multidisciplinary or "multi-disciplinary" or "multi-component" or multicomponent or "combined intervention" or "integrated care") and ("quality of life" or "health-related quality of life" or HRQoL or QoL or "EORTC

Database	Search_Strategy
	QLQ-C30" or "FACT-G" or "SF-36" or "SF-12" or "return to work" or RTW or "vocational rehabilitation") and (RCT or randomized or randomised or placebo)

Table S3. GRADE assessment of evidence certainty for primary outcomes

Table S3. GRADE assessment of evidence certainty for primary outcomes

Outcome	Trials	Patients	Effect	Certainty	Conclusion
Health-related quality of life (HRQoL)					
Exercise vs usual care	39	4,914	SMD 0.38 (0.26 to 0.50)	Moderate	Probably improves overall HRQoL
Psychoeducation vs usual care	13	1,845	SMD 0.40 (0.16 to 0.65)	Low	May improve overall HRQoL
Multidisciplinary vs usual care	12	1,133	SMD 0.27 (0.04 to 0.50)	Moderate	Probably improves overall HRQoL
Vocational vs usual care	3	273	SMD -0.09 (-0.43 to 0.25)	Low	May result in little to no difference
Return to work (RTW)					
Exercise vs usual care	4	631	OR 1.95 (0.99 to 3.87)	Moderate	May increase RTW rates
Psychoeducation vs usual care	6	1,978	OR 1.41 (1.15 to 1.73)	Moderate	Probably increases RTW rates

Table S3. GRADE assessment of evidence certainty for primary outcomes

Outcome	Trials	Patients	Effect	Certainty	Conclusion
Multidisciplinary vs usual care	3	325	OR 2.26 (1.15 to 4.48)	Low	May increase RTW, but evidence is imprecise
Vocational vs usual care	4	652	OR 1.29 (0.81 to 2.08)	Very low	The evidence is very uncertain

Supplementary Figures

Supplementary Figure S1. Forest plots of domain-specific health-related quality of life (HRQoL) by intervention type.

A. Exercise interventions were evaluated for role functioning, fatigue, emotional functioning, physical functioning, cognitive functioning, and social functioning;

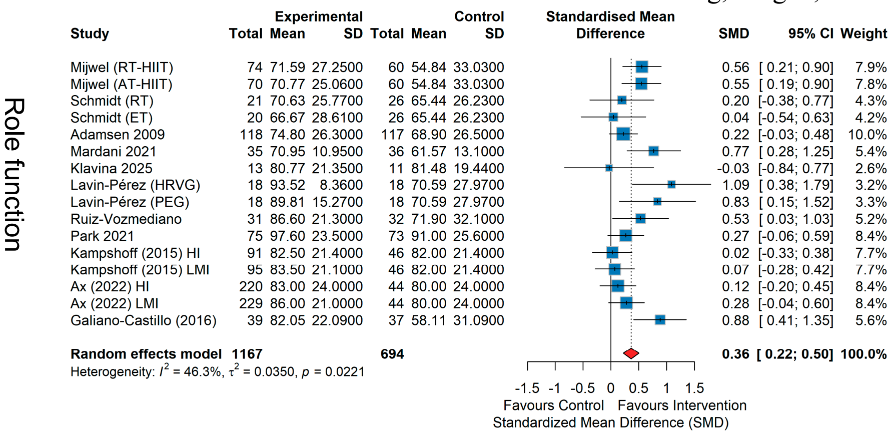

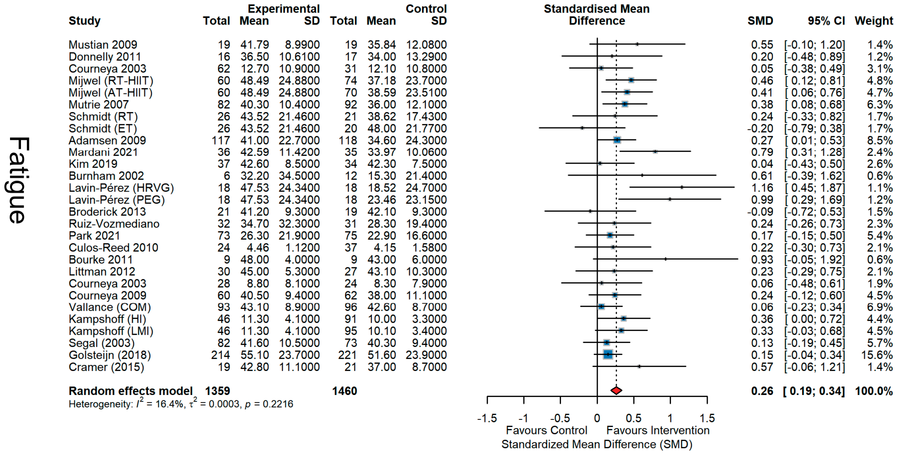

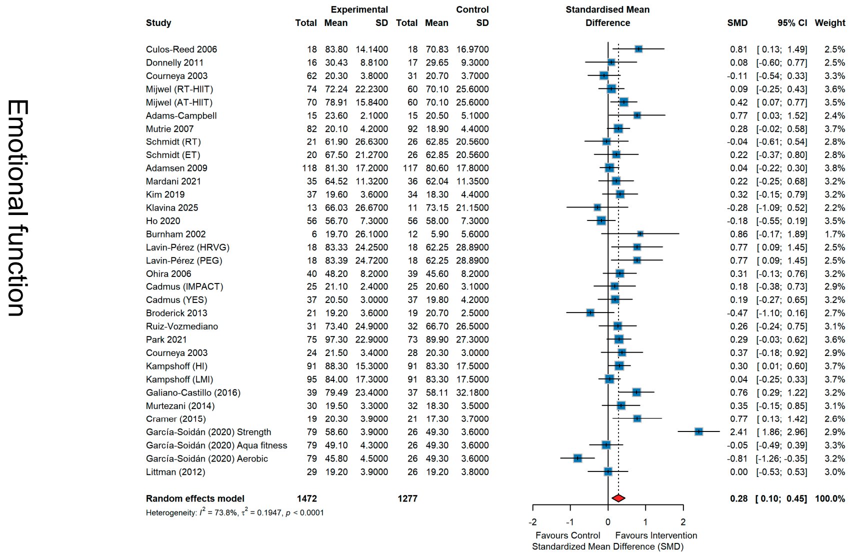

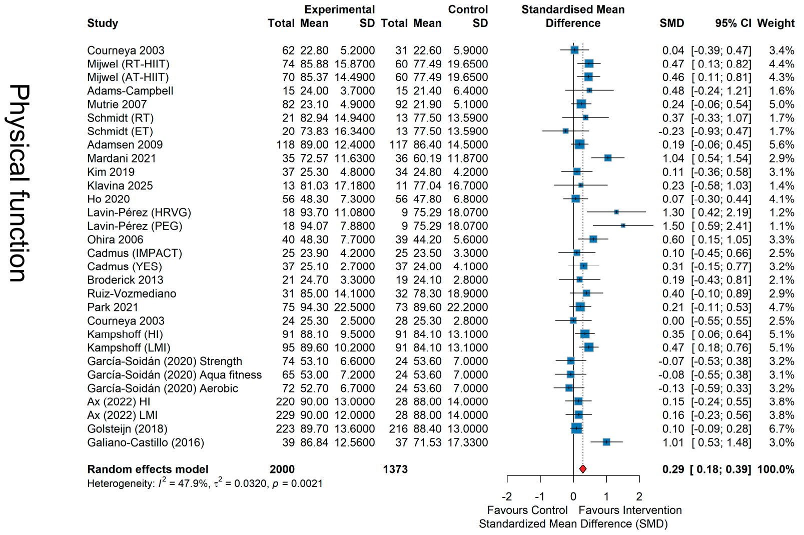

Cognitive function

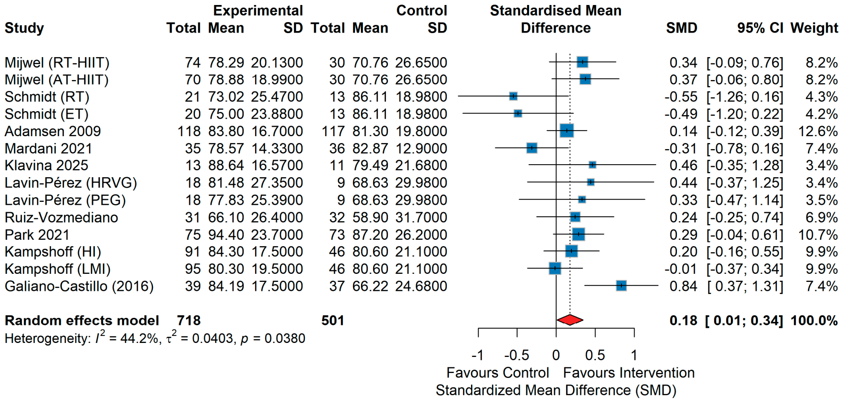

Social function

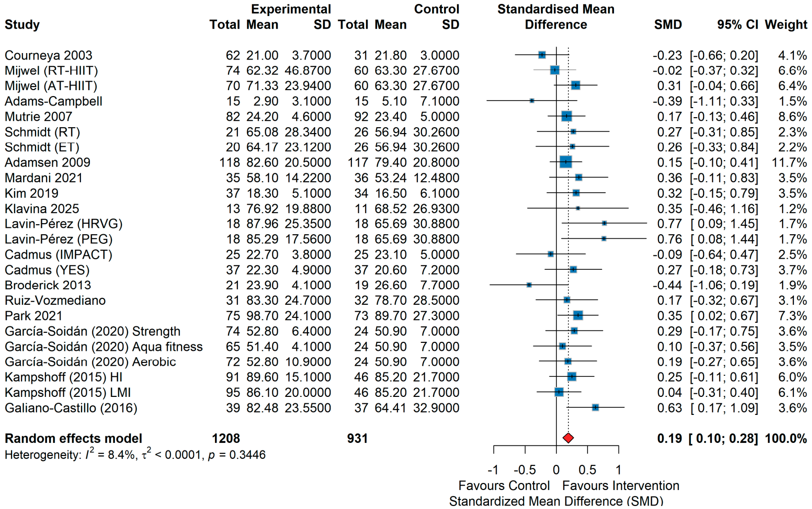

B. Psychoeducational interventions were evaluated for emotional functioning, physical functioning, and social functioning

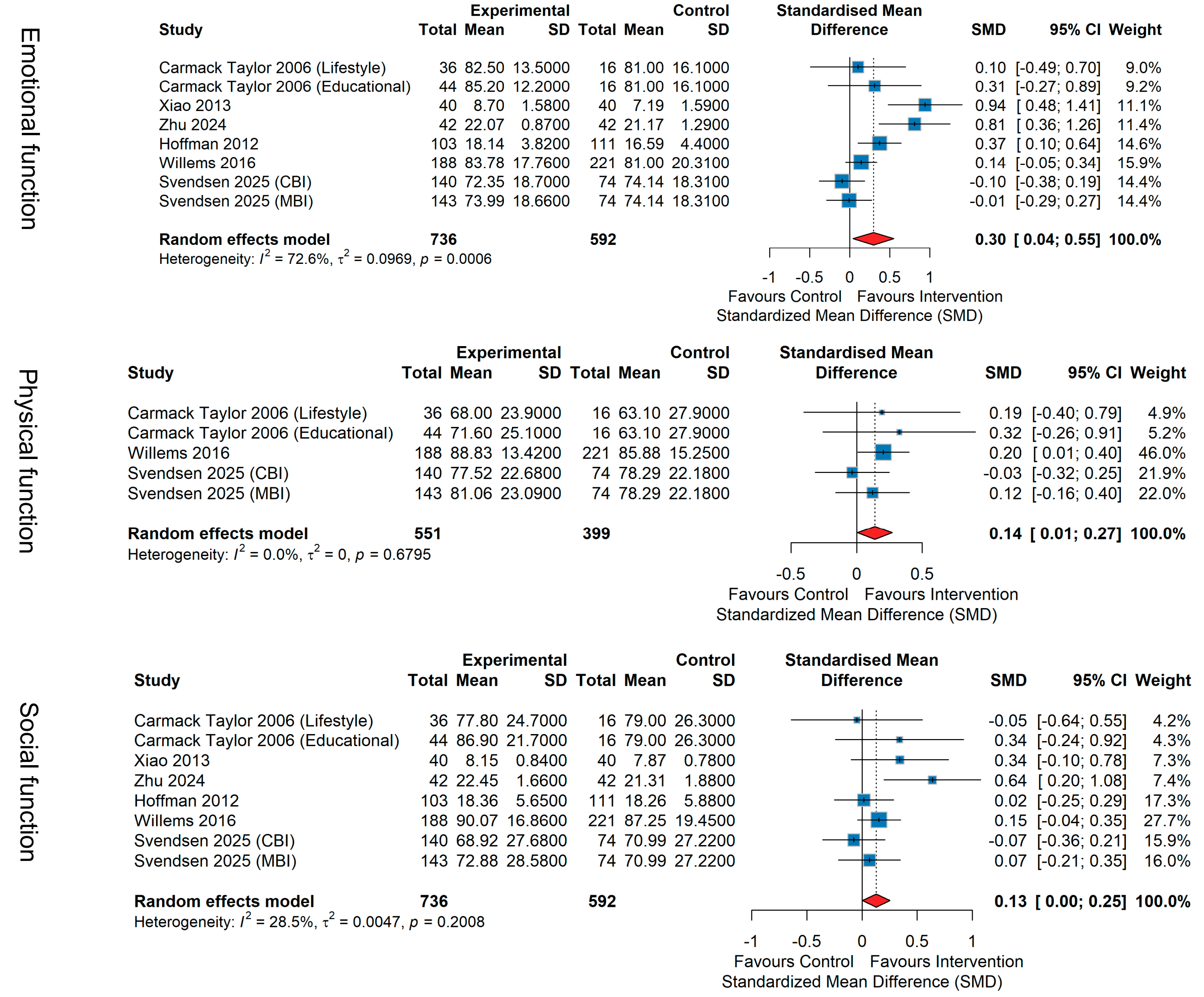

C. Vocational interventions were evaluated for role functioning, emotional functioning, physical functioning, and social functioning

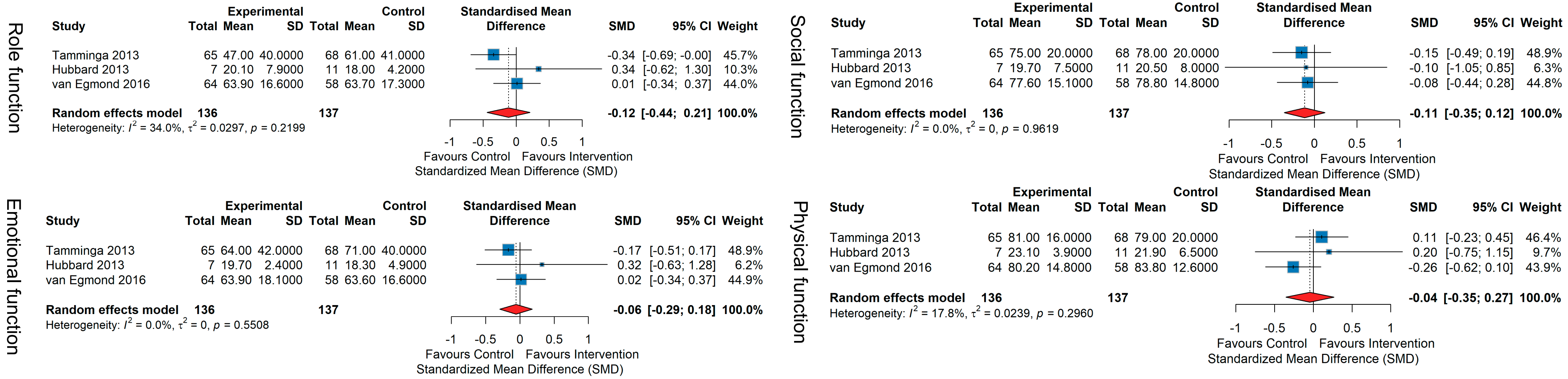

D. Multidisciplinary interventions were evaluated for role functioning, fatigue, emotional functioning, physical functioning, and social functioning

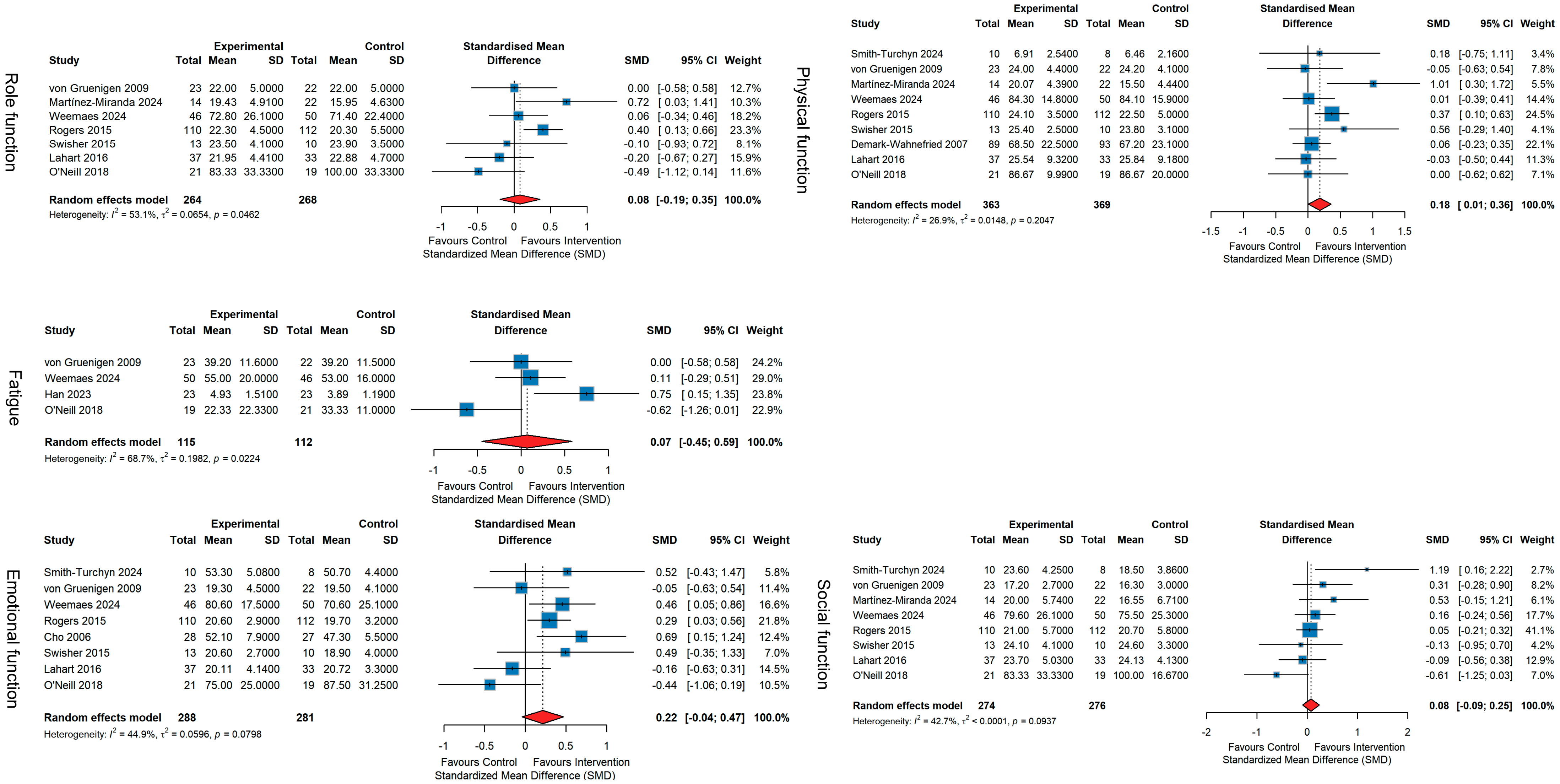

Figure S2. Leave-one-out sensitivity analyses of pooled effects by intervention type. Leave-one-out analyses were performed for overall health-related quality of life, return to work, and evaluable domain-specific health-related quality-of-life outcomes. Each point represents the pooled effect estimate after sequential exclusion of one study, with horizontal lines indicating 95% confidence intervals. Dashed vertical lines indicate the pooled estimate from the primary analysis. Colour gradients indicate the corresponding (I^2) value after each exclusion. HRQoL, health-related quality of life; RTW, return to work.

(A) Overall HRQoL

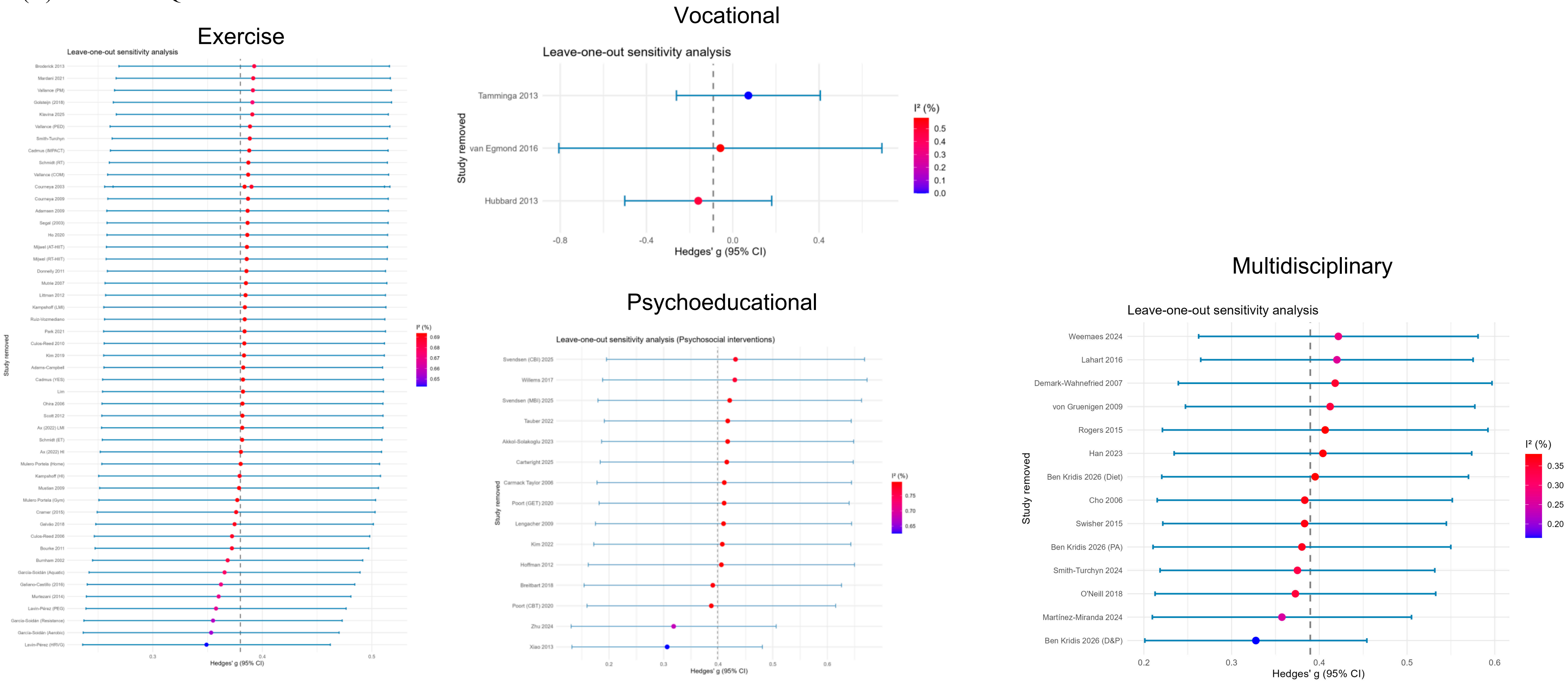

(B) RTW

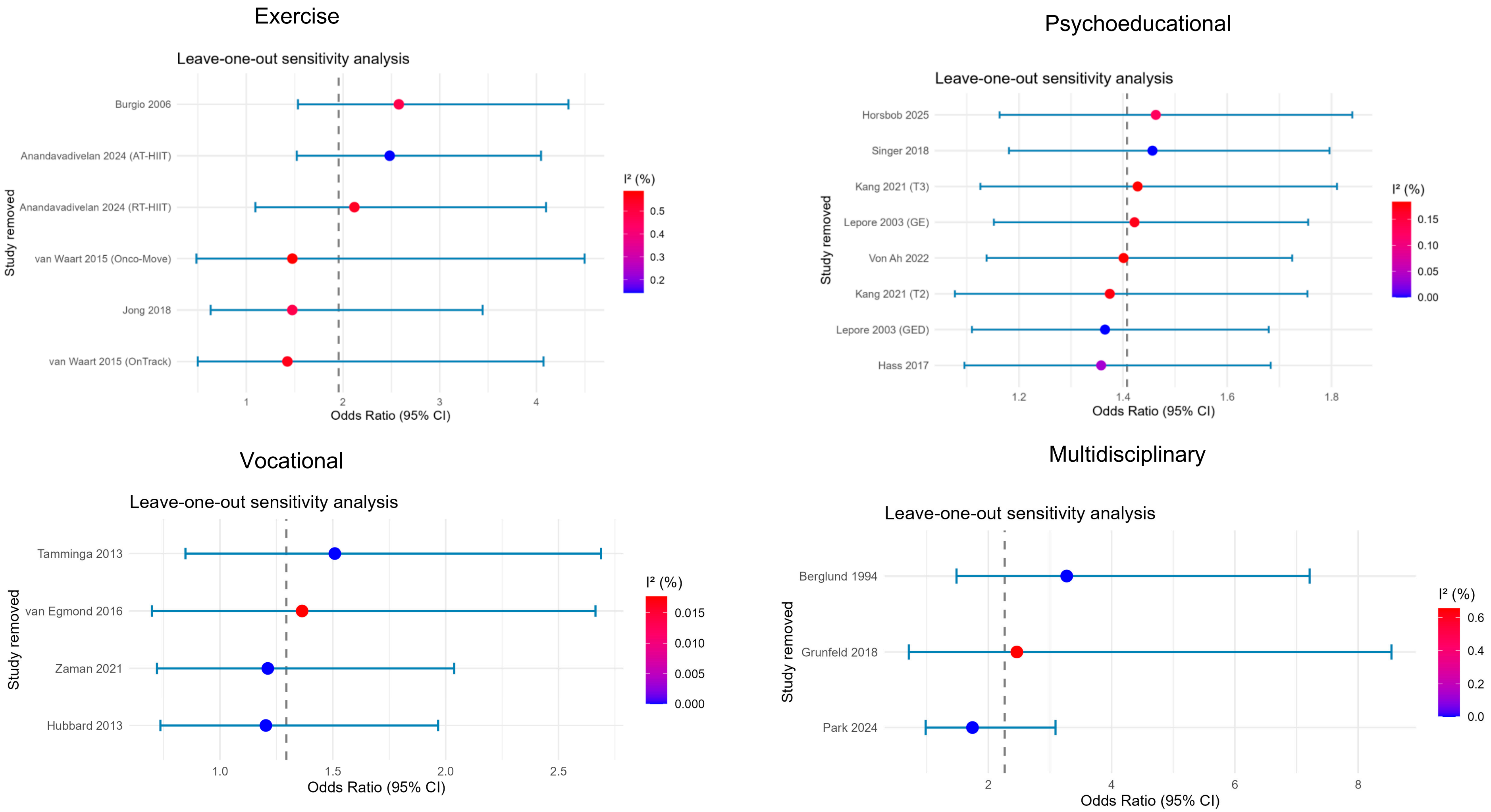

Exercise
Leave-one-out sensitivity analysis
Burgio 2006
Anandavadivelan 2024 (AT-HIIT)
Anandavadivelan 2024 (RT-HIIT)
van Waart 2015 (Onco-Move)
Jong 2018
van Waart 2015 (OnTrack)
Study removed
1
2
3
4
Odds Ratio (95% CI)
I² (%)
0.5
0.4
0.3
0.2
Psychoeducational
Leave-one-out sensitivity analysis
Horsbob 2025
Singer 2018
Kang 2021 (T3)
Lepore 2003 (GE)
Von Ah 2022
Kang 2021 (T2)
Lepore 2003 (GED)
Hass 2017
Study removed
1.2
1.4
1.6
1.8
Odds Ratio (95% CI)
I² (%)
0.15
0.10
0.05
0.00
Vocational
Leave-one-out sensitivity analysis
Tamminga 2013
van Egmond 2016
Zaman 2021
Hubbard 2013
Study removed
1.0
1.5
2.0
2.5
Odds Ratio (95% CI)
I² (%)
0.015
0.010
0.005
0.000
Multidisciplinary
Leave-one-out sensitivity analysis
Berglund 1994
Grunfeld 2018
Park 2024
Study removed
2
4
6
8
Odds Ratio (95% CI)
I² (%)
0.6
0.4
0.2
0.0

(C) Domain-specific HRQoL dimensions

C1.Exercise interventions were evaluated for role functioning, fatigue, emotional functioning, physical functioning, cognitive functioning, and social functioning;

Role function

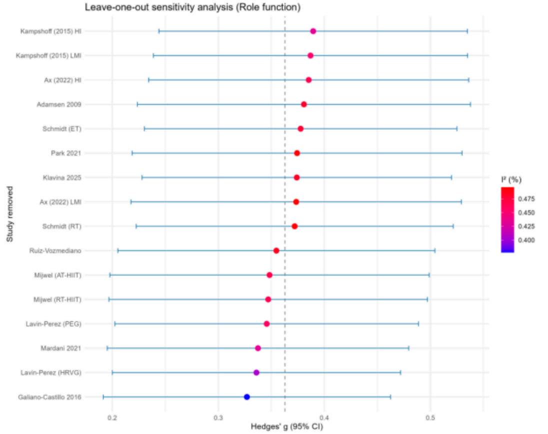

Fatigue

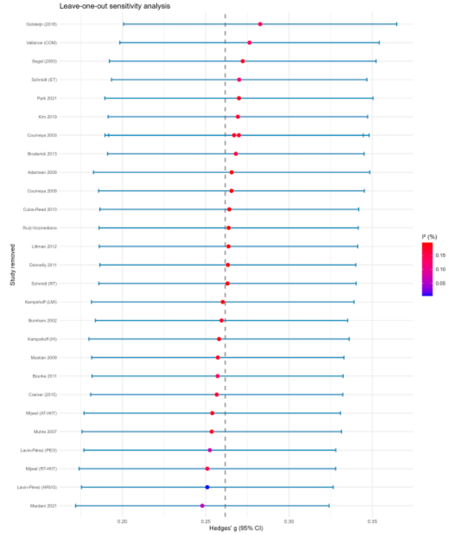

Physical function

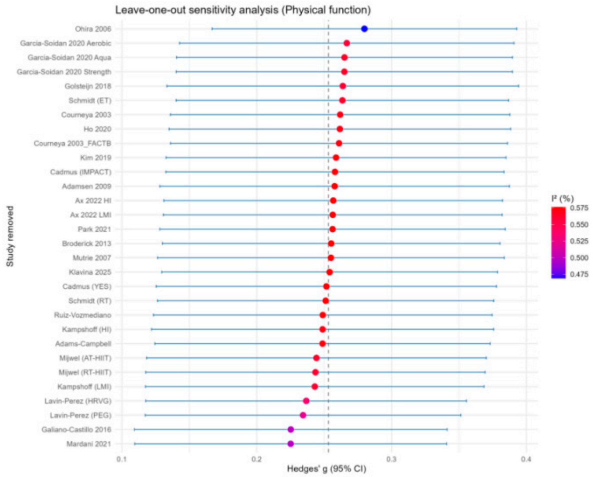

Emotional function

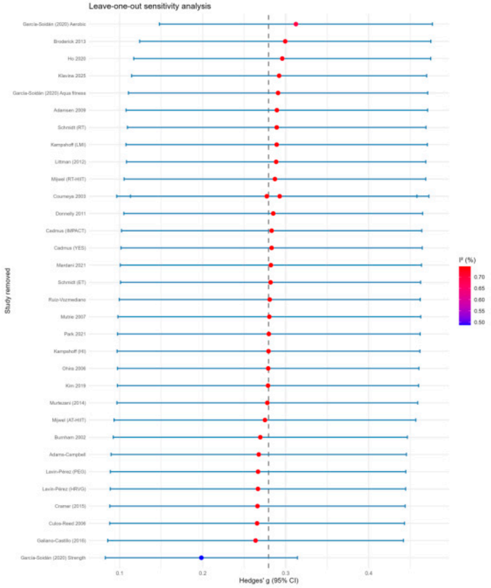

Cognitive function

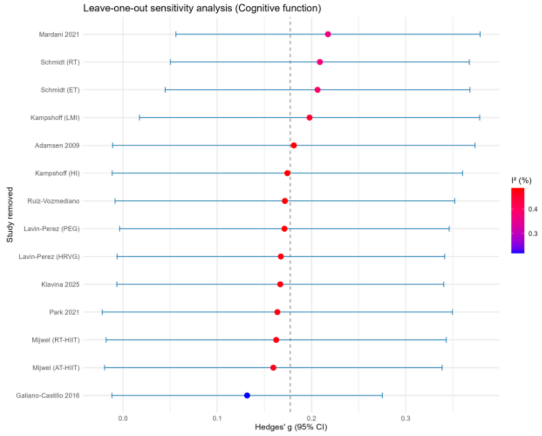

Social function

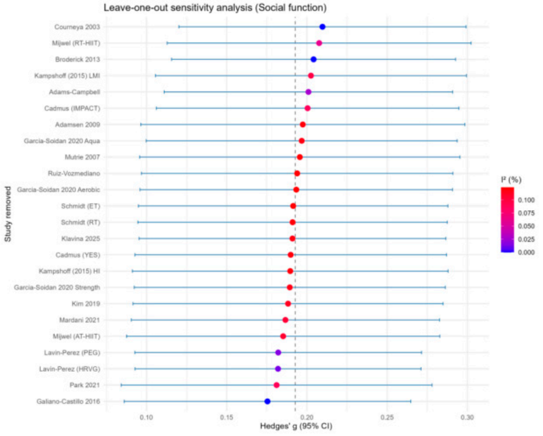

C2.Psychoeducational interventions were evaluated for emotional functioning, physical functioning, and social functioning

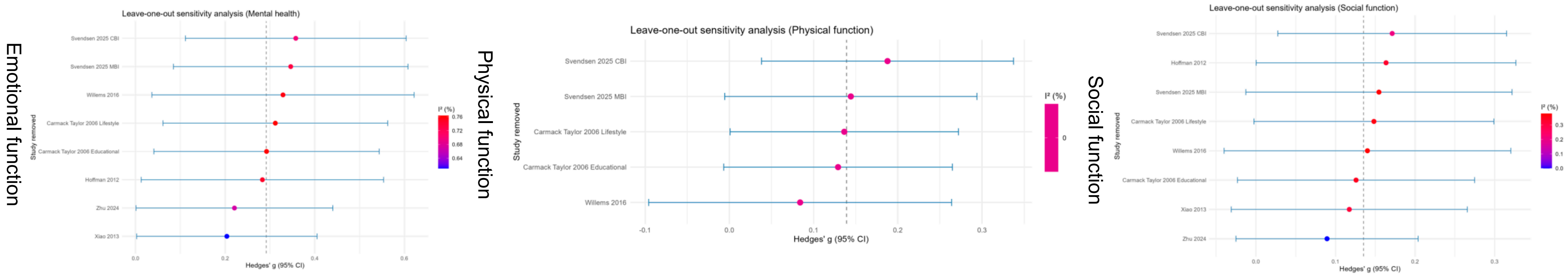

C3.Vocational interventions were evaluated for role functioning, emotional functioning, physical functioning, and social functioning

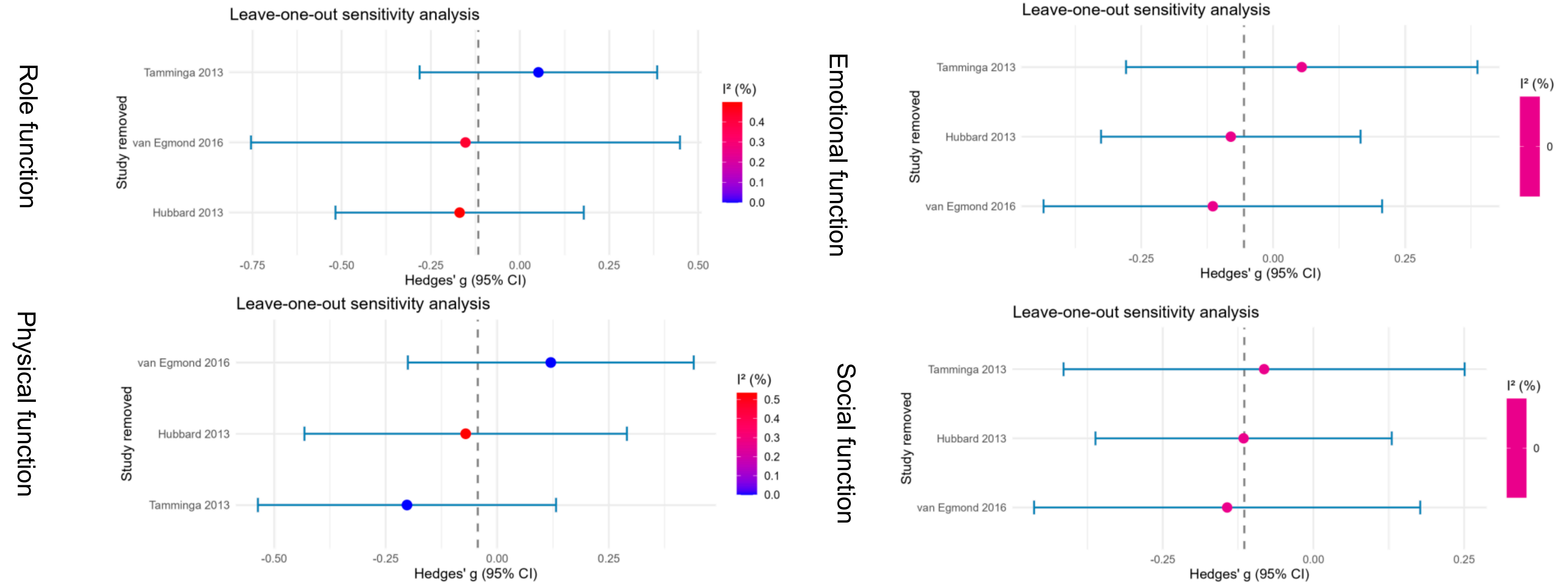

C4.Multidisciplinary interventions were evaluated for role functioning, fatigue, emotional functioning, physical functioning, and social functioning;

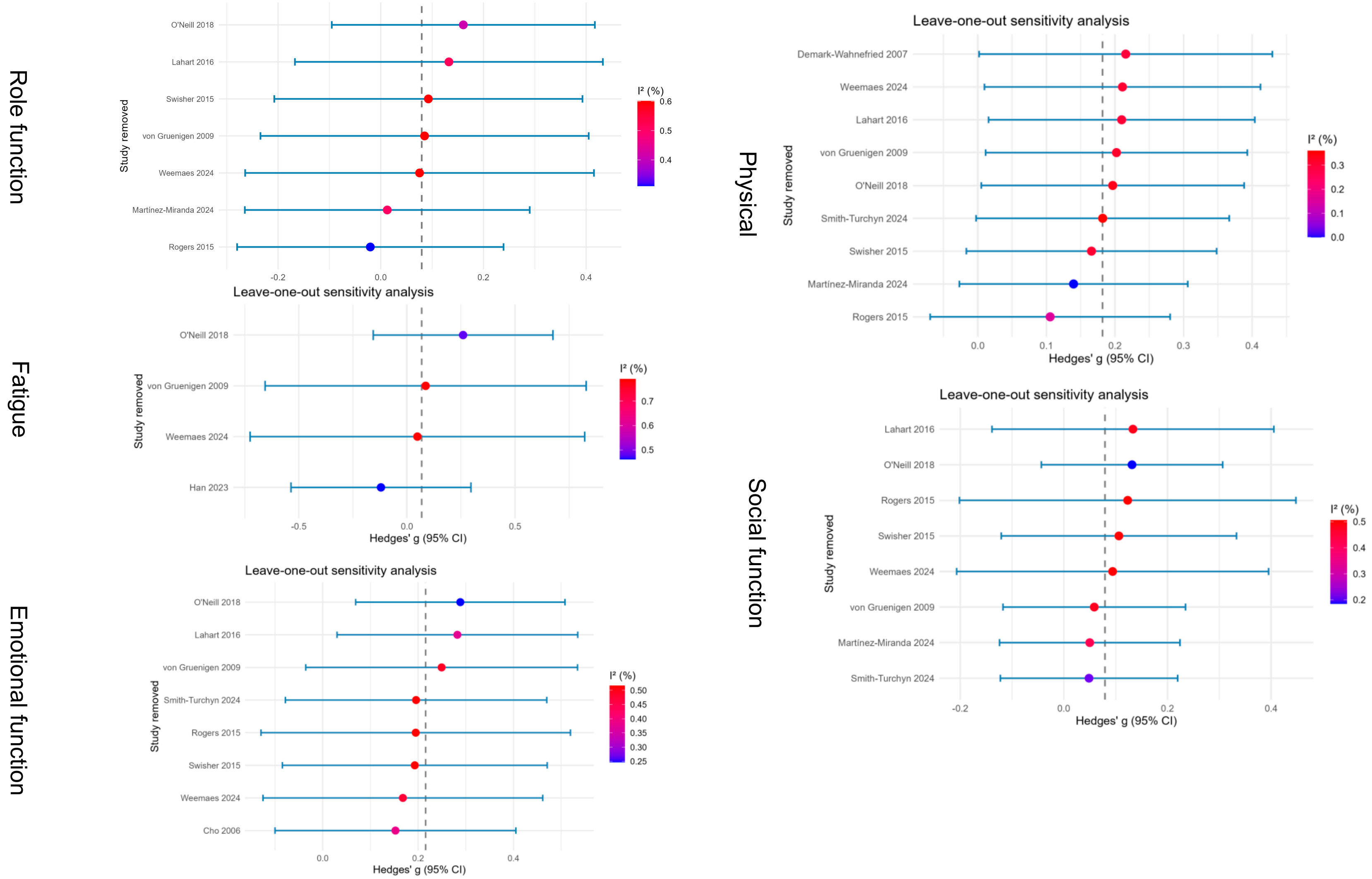

Figure S3. Funnel plots for publication bias assessment

(A) Funnel plots for overall HRQoL: Funnel plots for overall health-related quality of life (HRQoL) in exercise, psychoeducational, and multidisciplinary interventionss.

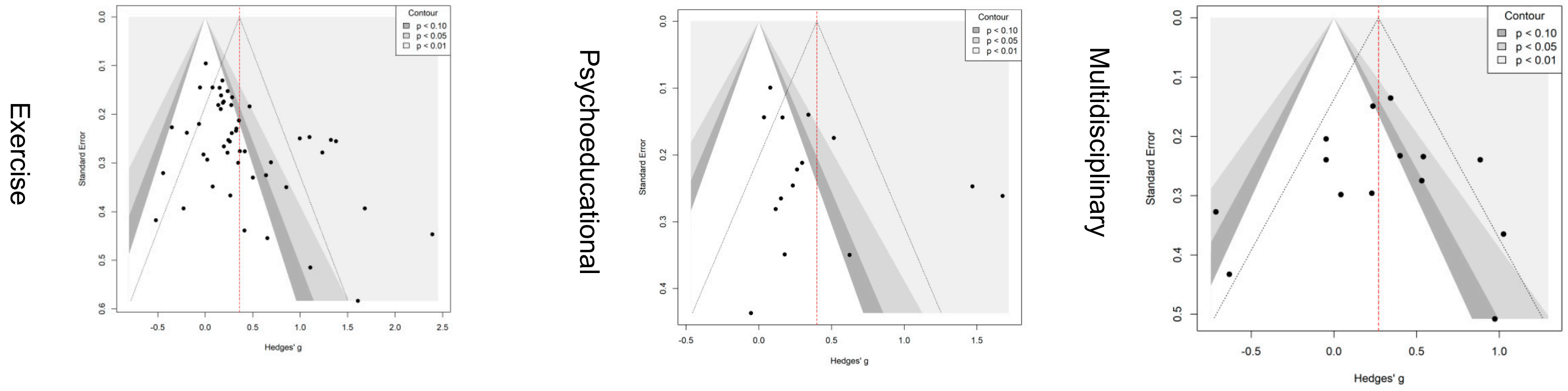

(B) Funnel plots for HRQoL domains in exercise interventions: Funnel plots for six HRQoL domains (role functioning, fatigue, emotional functioning, physical functioning, cognitive functioning, and social functioning) within exercise interventions.

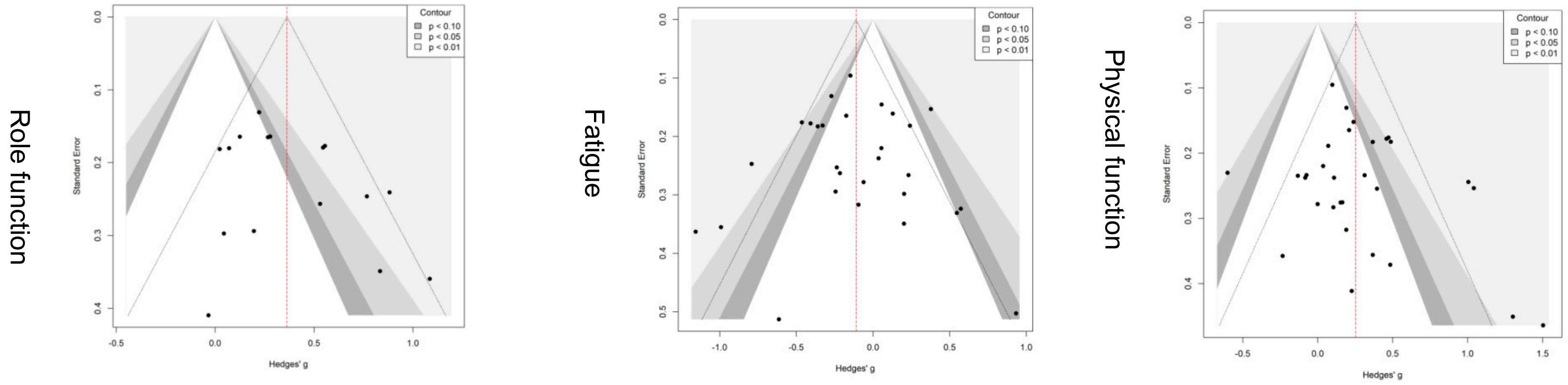

Cognitive functi

Emotional function

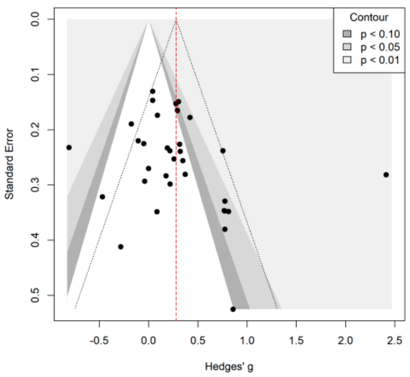

Cognitive function

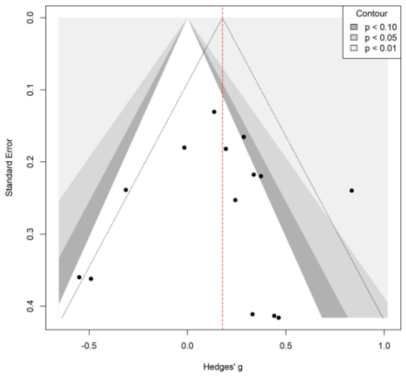

Social function

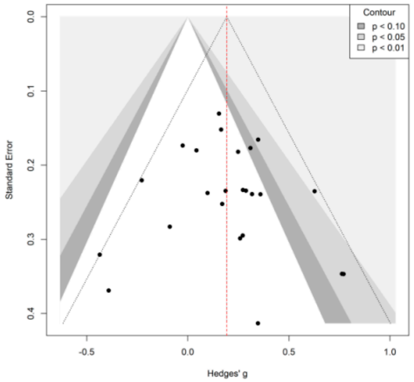

Supplement: Supplementary file 1 [file healthcare-14-03128-s001.zip › healthcare-4449874-supplementary.pdf]
